# Super-enhancer DNA methylation in cancer: the mechanism of action and therapeutic directions

**DOI:** 10.3389/fonc.2025.1610579

**Published:** 2025-10-01

**Authors:** Yue Bi, Kang Zou, Ke Wang

**Affiliations:** Department of Orthopedic Oncology, Yantaishan Hospital, Yantai, Shandong, China

**Keywords:** super-enhancer, DNA methylation, cancer, metastasis, tumor immunity, biomarker

## Abstract

The interplay between DNA methylation and super-enhancer activity is emerging as a key focus in cancer research. Super-enhancers, a specialized class of enhancers, strongly activate transcription of their target genes due to their dense clustering with essential transcription factors (TFs) and cofactors. In cancer, especially, these super-enhancers control key oncogenic drivers and often display abnormal DNA methylation patterns that can repress or overexpress target genes in both solid and blood cancers. Furthermore, DNA methylation in the super-enhancer region has been found to influence their regulatory capacity. Although enhancers are typically characterized by low DNA methylation, dysregulated methylation at super-enhancers is seen in most malignancies, affecting TF and chromatin regulator recruitment. Hypomethylation at these sites often accompanies oncogene hyperactivation, while hypermethylation can repress tumor suppressor mechanisms. Recent research highlights DNA methylation as a promising source of cancer biomarkers. This review examines the intricate relationship between DNA methylation and super-enhancer activity in cancer, concentrating on how methylation regulates super-enhancers, modulates oncogene expression, promotes oncogenesis, and serves as a target for novel oncology therapies.

## 1 Introduction

A specialized class of enhancers, termed super enhancers, has garnered significant attention due to their heightened transcriptional activity and implications in cellular identity and pathology (1). Compared to typical enhancers, super enhancers exhibit enhanced recruitment of the regulatory molecules, leading to robust transcriptional activation of target genes (2). Super enhancers are linked to essential developmental regulating genes, and their roles are altered in cancer (3). Of note, epigenetic modifications are central to the function and maintenance of super enhancers (4). DNA methylation, an epigenetic modification involving the addition of methyl groups to the cytosine bases of CpG dinucleotides, is a key regulator of super-enhancer activity (4). However, aberrant DNA methylation of super-enhancers—either hypermethylation or hypomethylation—has been increasingly recognized as a hallmark of cancer (5). These epigenetic alterations impact the activity of super-enhancers, leading to the silencing of tumor suppressor genes or activation of oncogenes, thereby promoting tumor initiation and progression (6). For example, studies on head and neck squamous cell carcinomas (HNSCC) and breast cancer show that hypermethylated super-enhancers are associated with reduced expression of genes critical for cellular homeostasis, resulting in the overexpression of oncogenic drivers, enhancing tumorigenic traits such as proliferation, invasion, and angiogenesis (7–9). Of note, human malignancies show distinctive DNA methylation alterations, such as genome-wide hypomethylation and site-specific hypermethylation (10, 11).

The relationship between DNA methylation and super-enhancers can also be explored through functional genomics findings (12, 13). In this sense, Heyn et al. (13) evaluated the methylation patterns of over 5,000 super-enhancers across normal tissues, primary tumors, and metastatic samples. Their findings revealed a distinct alteration in the DNA methylation profiles of super-enhancers in cancer compared to healthy controls. Specifically, reduced methylation in super-enhancers was consistently associated with increased gene expression, whereas increased methylation was correlated with decreased expression levels (13). Additionally, Chen et al. (14) found that the expression of super-enhancer RNA and CpG methylation are both pivotal in the progression of The Cancer Genome Atlas (TCGA) melanoma and other cancers. Their study found the critical role of CpG DNA methylation in regulating super-enhancers and their associated eRNA loci during tumorigenesis (14). By analyzing eRNA loci across multiple cancer types, significant methylation changes were identified in 1,187 CpG dinucleotides, with distinct clusters of hypermethylation and hypomethylation correlating with eRNA activation or deactivation (14). These alterations were shown to affect eRNA expression in 360 loci, with hypermethylation linked to eRNA locus deactivation and hypomethylation associated with activation, highlighting the epigenetic regulation of super-enhancers in cancer progression (14). This review explores the multifaceted roles of DNA methylation in modulating super-enhancer activity in cancer. Additionally, we discuss the potential for targeting super enhancer-associated DNA methylation as a therapeutic strategy in cancer.

## 2 The definition of super enhancer, DNA methylation, and regulatory mechanisms

Super-enhancers are a specialized group of regulatory factors that have been defined in terms of size, density of transcription factor binding, and capacity to control very high levels of gene transcription (15). Initially described as enhancer clusters with tight binding to transcriptional coactivators, such as Mediator complex subunit 1 (MED1) and bromodomain-containing protein 4 (BRD4), super-enhancers are now defined as key nodes that play significant roles in cell identity and disease-associated gene regulation (15). A number of epigenetic signatures distinguish super-enhancers from typical enhancers. Histone marks are among the most widely used markers: Super-enhancers have elevated levels of H3K27ac and H3K4me1 marks, characteristic marks of active enhancer arrangements, and their function declines when these marks are reduced. Super-enhancers also generate enhancer RNAs (eRNAs), brief non-coding reads bidirectionally transcribed from enhancer elements (2). eRNAs are suspected to stabilize enhancer–promoter looping, attract chromatin remodelers, and promote transcriptional activation of target genes. Moreover, some lncRNAs engage with super-enhancers to function as platforms for transcriptional complexes, regulate chromatin structure, and confer tissue-specific regulatory specificity (16). Epigenetic modifications in the form of DNA methylation also characterize super-enhancers from typical enhancers (13). Super-enhancers tend to exhibit lower levels of 5-methylcytosine (5mC), a feature that maintains chromatin accessibility and facilitates transcription factor binding (17). In contrast, abnormal hypermethylation of super-enhancers can suppress tumor-suppressor pathways, whereas hypomethylation can cause overexpression of oncogenes. The most recent research also identifies 5-hydroxymethylcytosine (5hmC) as a super-enhancer regulator, indicating that active DNA demethylation is involved in enhancer plasticity (18). Collectively, the signature characteristics of super-enhancers are (1): widespread occupation by transcription factors and coactivators (2), active histone mark density including H3K27ac (3), eRNA production and lncRNA participation in enhancer-promoter contacts, and (4) dynamic DNA methylation landscapes to manage enhancer function. These characteristics enable super-enhancers to function as master regulators of gene expression programs, distinguishing them from typical enhancers and highlighting their significance in cancer biology.

**Figure 1:**
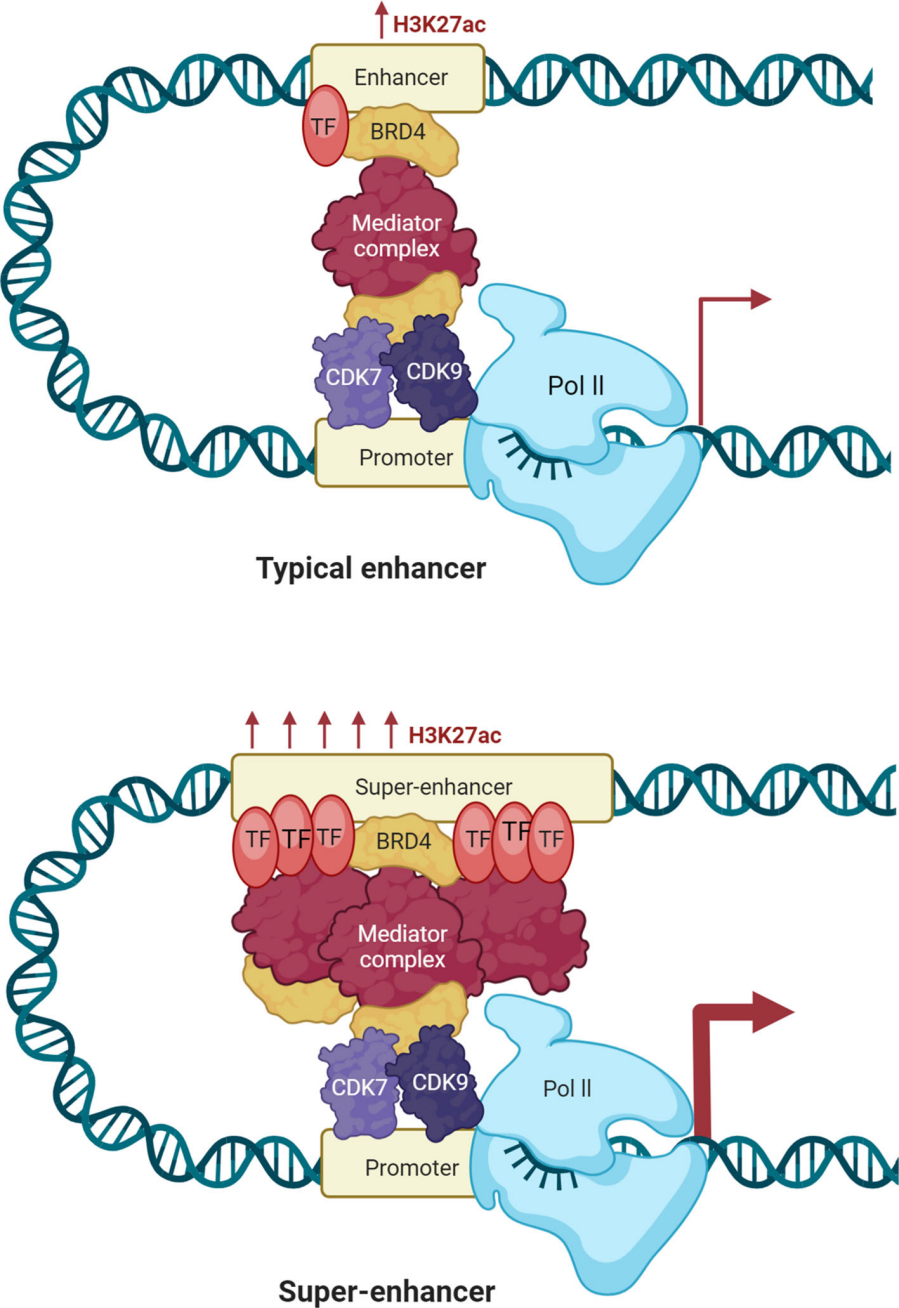
Enhancers and super-enhancers in gene regulation. Enhancers are short DNA regions that act as gene activators of transcription by bringing activator proteins, such as transcription factors. Enhancers regulate gene expression in space and time during development by either acting in cis (local) or trans (distal). Active enhancers have H3K4me1- and H3K27ac marks, and poised enhancers have H3K4me1 marks but no H3K27ac marks. Enhancers are depleted of nucleosomes, deficient in H3K4me3, and hypersensitive to DNase I. Enhancers interact with target promoters by chromatin looping, facilitated by cofactors and mediators, and have the capacity to recruit RNA Polymerase II (RNAPII) to transcribe enhancer RNAs (eRNAs). eRNAs enable transcriptional activation by stabilizing enhancer–promoter looping and regulating recruitment of transcription factors. DNA methylation also controls enhancer accessibility, with low levels retaining enhancer function, but disease-causing hypermethylation suppressing enhancer function. Super-enhancers are longer sets of enhancers with extremely high levels of coactivator and transcription factor binding. They are enriched in H3K4me1 and H3K27ac, with low levels of DNA methylation that retain chromatin accessibility, and high levels of eRNA to enhance their regulatory effect. Super-enhancers will be present in CTCF- and cohesin-maintained chromatin loops within topologically associating domains (TADs). Integrating histone marks, DNA methylation profiles, and eRNA expression, super-enhancers are the supervisors of transcription programs, most prominently those defining cell identity or initiating oncogene expression.

Super-enhancers are defined as a category of regulatory regions characterized by a significant enrichment for the binding of transcriptional coactivators, particularly Med1 (**Figure 1**) (19, 20). Regions associated with key regulators like octamer-binding transcription factor 4 (Oct4), SRY-Box Transcription Factor 2 (Sox2), and Nanog, identified through chromatin immunoprecipitation sequencing (ChIP-seq), were categorized as enhancers (19, 20). Enhancers within 12.5 kb of one another were merged into a single genomic unit, and both these merged units and standalone enhancers without neighboring elements in this range were ranked according to the total Med1 signal normalized to background levels in their respective genomic regions (21). A minor fraction of these enhancer regions exhibited Med1 levels exceeding a threshold established by analyzing the distribution of ChIP-seq intensity values, with enhancer regions to the right of the point where the slope of the plot equaled 1 classified as super-enhancers (22). Super-enhancers regulate the expression of genes that determine cell identity and are crucial for the specific biological processes (22). In tumorigenesis, genes associated with super-enhancers are crucial for sustaining cancer cell identity and facilitating oncogenic gene transcription (19, 22).

DNA methylation involves the addition of a methyl group to the carbon-5 of the cytosine base, resulting in 5mC, which predominantly occurs within cytosine-guanine dinucleotides (CpG) due to the activity of DNA methyltransferase enzymes (DNMTs) (23). The significant interest in DNA methylation stems from its essential functions in cell biology, including the regulation of gene expression, silencing of retroelements, chromosome segregation during mitosis, X-chromosome inactivation, and monoallelic silencing of imprinted genes (23).

DNA methylation plays a pivotal role in regulating the functionality of super-enhancers, which are distinguished by their significantly higher GC content compared to conventional enhancers and are highly sensitive to methylation dynamics (24). Interestingly, the relationship between DNA methylation and super-enhancer activity is not uniform across all loci but can vary within the same genomic region. This variability has been observed in embryonic stem cells and epiblast stem cells, where differences in methylation levels correlate with distinct super-enhancer activity patterns. Notably, the genes regulated by these methylation-dependent super-enhancers are often associated with maintaining the naïve state of pluripotency, underlining their importance in early developmental processes (25). Further studies, including those conducted by Song et al. (26), clarified the mechanism through which DNA methylation regulates chromatin states. Specifically, DNA methylation regulates H3K27ac, a histone modification critical for super-enhancer activity, through an interplay between DNA methyltransferases and transcription factors. This balanced interaction underscores the complex epigenetic crosstalk that governs gene regulation via super-enhancers.

## 3 Oncogenic super-enhancer function

In cancer, specific super-enhancers, termed oncogenic super-enhancers, emerge within tumor cells to enhance oncogene expression, leading to the dysregulation of key pathways and driving malignancy (27). Initially identified in multiple myeloma cells, oncogenic super-enhancers exhibit high binding densities of transcriptional coactivators like MED1 and BRD4 and have since been implicated in a variety of cancers (20). Recent studies have identified several oncogenic super-enhancers in cancers such as small-cell lung cancer (SCLC), neuroblastoma, esophageal cancer, colorectal cancer (CRC), and melanoma (22, 28). Oncogenic super-enhancers contribute to cancer progression through several mechanisms, including the activation of pathways like mitogen-activated protein kinase (MAPK), which inhibits apoptosis and enhances proliferation (29). Super-enhancers drive the elevated expression of the erythroblastosis virus E26 oncogene homolog (ERG), which in turn activates target genes that contribute to cancer progression (30). Oncogenic super-enhancers have been shown to upregulate the expression of genes like *GJA5, CYP24A1, SLAMF7*, and *ETV145* (31). In adenoid cystic carcinoma, nuclear translocation of super-enhancers drives increased MYB expression, while in pheochromocytomas and paragangliomas, super-enhancers play a key role in promoting TERT expression (32). ChIP-seq evaluation of CRC indicated that transcription factor 4 (TCF4) functions as a terminal transcription factor in the Wnt pathway and is located at the c-MYC locus (33). TCF4 is a target of Wnt, exhibiting a pronounced H3K27Ac signal following the acquisition of oncogenic super-enhancers by cancer cells (33). ChIP-seq evaluation of H3K27Ac in MCF-7 cells revealed that the super-enhancer targeting the *ESR1* gene, which encodes the estrogen receptor alpha (ERα) exclusively, regulates genes enriched in processes associated with ERα binding in estrogen receptor-positive breast cancer cells (34). Super-enhancers play a pivotal role in channeling oncogenic signaling pathways into gene expression programs that are critical for sustaining cancer progression (33). Certain super-enhancers are frequently identified in CRC samples (35). The assignment of super-enhancers to adjacent genes revealed a subset of genes associated with malignancy that are regulated by super-enhancers. The expression of interleukin (IL)-20 receptor alpha (RA) is dysregulated through enhancer hijacking, a mechanism involving chromosomal rearrangements that reorganize super-enhancers, leading to oncogenesis (36). Profiling of H3K27ac in lung adenocarcinoma (LUAD) cells versus normal lung tissues demonstrated the presence of cancer-specific and normal-specific super-enhancers (37). Cancer-specific super-enhancer target genes were found to be enriched in LUAD driver genes and tumor signaling pathways, whereas normal-specific super-enhancer target genes were associated with immune functions (38). The homeobox B (HOXB) cluster locus, commonly associated with super-enhancers, contains a super-enhancer present in primary CRC tissues but absent in normal colon tissues, highlighting its specificity to CRC (39). HOXB overexpression was essential for sustaining malignant phenotypes in CRC and is regulated by the super-enhancer associated with the HOXB cluster. Furthermore, multiomic profiling of the super-enhancer landscape in triple-negative breast cancer demonstrated that cells within the same subtype exhibited a greater degree of super-enhancer similarity (40). Specific side effects of triple-negative breast cancer are linked to various oncogenes such as *MET, FOXC1*, and *MYC* (40). Taken together, these findings underscore the critical role of oncogenic super-enhancers in shaping cancer phenotypes and driving malignancy (**Table 1**).

**Table 1:** Role and function of super-enhancer in cancer.

Cancer	Study	Mechanism	Gene	Function	Ref.
Multiple myeloma (MM)	*In vitro*	BRD4	*RUNX1*, *BCL3* and *FOSL2*	In MM, key factors influencing the tumor state were linked to extensive enhancer regions known as super-enhancers. These regions exhibited unusually high concentrations of Bromodomain Containing 4 (BRD4) and Mediator complexes.	(20)
T-cell acute lymphoblastic leukemia (T-ALL)	Clinical and *in vitro*	H3K27ac	*TAL1*	The majority of endogenous super-enhancers in T-ALL cells were enriched with MYB and CBP, indicating that MYB likely plays a central role in initiating super-enhancer formation.	(41)
Diffuse large B cell lymphoma (DLBCL)	*In vivo*	BbD4	*MYC* and *E2F*	A functional analysis of genes associated with super-enhancers revealed that DLBCLs rely on OCA-B, highlighting a potential approach for identifying previously unrecognized cancer dependencies.	(42)
Erythroleukemia	*In vitro*	LSD1, CoREST, HDAC1, and HDAC2	*GFI1*	Analysis using gene set enrichment demonstrated that removing the GFI1 super-enhancer disrupted pathways driven by NCD38, which are associated with granulocyte differentiation and the CEBPA network. Conversely, it reactivated pathways suppressed by NCD38, including those involved in erythroid development, GATA1-regulated targets, and specific acute myeloid leukemia (AML) clusters, such as FAB subtype M6 and AML linked to chromosomal abnormalities associated with myelodysplastic syndromes.	(38)
Medulloblastoma	*In vivo*	BRD4	*ALK, SMO and NTRK3, LMO1, LMO2, MYC*, *ETV4* and *PAX5*	Super-enhancers in medulloblastoma were found to regulate key genes listed in the Cancer Gene Census. These include ALK in the WNT subgroup; SMO and NTRK3 in the SHH subgroup; LMO1, LMO2, and MYC in Group 3; and ETV4 and PAX5 in Group 4.	(43)
Neuroblastoma	*In vitro*	cyclin-dependent kinase 7 (CDK7)	*MYCN*	Super-enhancers played a critical role in driving the elevated expression of oncogenic MYCN. The overexpressed MYCN protein extensively interacted with promoter and enhancer regions across the genome, including its own regulatory elements, resulting in widespread transcriptional activation.	(44)
Neuroblastoma	*In vitro*	MYB	*LMO1*	The study revealed that a polymorphism within a super-enhancer element located in the first intron of LMO1 impacts neuroblastoma susceptibility by altering GATA transcription factor binding. This, in turn, directly regulates LMO1 expression in cis, creating an oncogenic dependency in the tumor.	(45)
Glioblastoma	*In vitro*	MAPK/ERK	*CDK6, SOX2, EGFR* and *BRD4*	Super-enhancers were shown to influence key genes involved in glioblastoma stem cell identity, development, and therapeutic resistance. The study emphasized the connection between chromatin landscapes and gene expression profiles, revealing how super-enhancers drive the transcriptional programs that sustain glioblastoma’s aggressive and heterogeneous nature.	(46)
Small-cell lung cancer	*In vitro*	CDK7	*MYC, SOX2*, *OTX2* and *NFIB*	The proto-oncogenes C-MYC (in GLC16 and NCI-H82 cells) and MYCN (in NCI-H69 cells), which are locally amplified, were found to be linked to extensive super-enhancers and showed significant sensitivity to THZ1 treatment.	(47)
Lung adenocarcinoma (LUAD)	Clinical and *in vitro*	–	*PSMB5* and *TOP2A*	Through analysis of key super-enhancers, two unique subtypes were identified, each displaying distinct patterns of genomic alterations (like mutations and variations in copy number) and corresponding differences in clinical prognosis.	(37)
Breast cancer	*In vitro*	CDK7	*SMAD3*, *TCF7*, *STAT3*, *CTCF*	The constituent enhancers of super-enhancers that regulate genes in the Achilles cluster in Triple-negative breast cancer (TNBC) cells were found to be enriched with DNA-binding motifs recognized by signaling transcription factors.	(34)
Breast cancer	*In vitro* and *in vivo*	ANLN	*FOXC1* and *MET*	A mechanism has been identified through which ANLN was upregulated in TNBC, with these findings highlighting the clinical and biological importance of the ANLN super-enhancer in the tumorigenesis of TNBC.	(40)
Oesophageal squamous cell carcinoma (OSCC)	*In vitro* and *in vivo*	CDK7	*PAK4, RUNX1, DNAJB1, SREBF2 YAP1*, and *PAK4*	Several lineage-specific master regulators in OSCC, and an integrative analysis of THZ1-sensitive and super enhancer-associated transcripts, uncovered a set of novel oncogenes in *OSCC*, such as *PAK4*, *RUNX1*, *DNAJB1*, *SREBF2*, and *YAP1*. Notably, PAK4 emerged as a potentially targetable kinase for therapeutic intervention.	(48)
Neck and nasopharyngeal squamous cell carcinoma	*In vivo*	BRD4, NF-κB p65	*ETV6, MET, TP63* and *FOSL1*	Targeting super-enhancers with BET inhibitors proved to be an effective strategy for inhibiting the growth and metastasis of HNSCC, as it simultaneously eradicated cancer stem cells (CSCs) and the mitotic bulk of the tumor.	(49)
Colon cancer	*In vitro*	mitogen-activated protein kinase (MAPK)	*BRAF*	Super-enhancers specific to colon cancer were found to be linked to various oncogenic pathways, with a notable association with the MAPK pathway.	(29)
Colorectal Cancer (CRC)	Clinical and *in vitro*	–	*IL-20RA*	IL-20RA has been identified as a key regulator of oncogenic signaling and immune dynamics in CRC, driving the expression of genes that promote tumor progression, unchecked cell division, and evasion of immune surveillance.	(36)
Gastric adenocarcinoma	Clinical and *in vitro*	CDX2 and HNF4α	*ABHD11, CLDN3* and *CLDN4*	Patients with gastric cancers characterized by elevated expression of genes linked to predicted super-enhancers experienced significantly worse overall survival compared to those with lower expression levels of these genes.	(50)
Hepatocellular carcinoma (HCC)	Clinical and *in vitro*	CDK7, BRD4, EP300, MED1	*SPHK1, MYC, MYCN, SHH*, and *YAP1*	HCC cells displayed exceptional sensitivity to super-enhancer complex disruption, primarily due to the selective inhibition of oncogenes driven by these regulatory elements.	(51)
Melanoma	*In vivo*	INO80	*SOX10* and *AXL*	INO80 binding showed a substantial overlap and strong positive correlation with key enhancer and super-enhancer markers, including H3K4me1, H3K27ac, and Med1. These findings collectively indicate that INO80 directly interacts with super-enhancers to drive the expression of oncogenic genes in melanoma.	(52)
Ewing sarcoma	*In vitro*	cyclin D1/CDK4	*EWS/FLI*	The research identified a super-enhancer as a key regulator of the *cyclin D1* gene (CCND1) and highlighted the selective reliance of Ewing sarcoma on *CCND1* and *CDK4*, setting it apart from other types of cancer cell lines.	(53)
Pancreatic cancer	*In vivo*	KDM6A	*ΔNp63, MYC*, and *RUNX3*	Loss of KDM6A specifically promoted the development of squamous-like, metastatic pancreatic cancer in females by disrupting the COMPASS-like complex and aberrantly activating super-enhancers that regulate key oncogenes, including *ΔNp63, MYC*, and *RUNX3*.	(54)

## 4 Super-enhancer DNA methylation in various aspects of cancer

Super-enhancers are important regulatory factors that control genes required for cell identity and functional maintenance (22). However, in cancer, the epigenetic landscape of super-enhancers is frequently altered, particularly through DNA methylation (23). These aberrant methylation patterns profoundly affect gene regulation, contributing to cancer initiation, metastasis, and resistance. In this section, we overview and discuss the latest data on the role and action mechanism of super-enhancer DNA methylation in cancer.

### 4.1 Oncogenic overexpression of adhesion molecules

DNA methylation of super-enhancers is crucial for the regulation of oncogene and adhesion molecule expression in cancer (55). Aberrant methylation of super-enhancer regions can result in the dysregulation of gene expression, affecting oncogenic pathways and adhesion molecules. Choudhury et al. (55) described that DNA methylation of adhesion-related genes is dynamically variable in a variety of myeloma (MM) subgroups according to solidly established methylation-expression correlations, suggesting that gene-level analyses would be needed to elucidate how DNA methylation operates in concert with other epigenetic modulators of gene expression. In the present study, oncogenic cMAF expression was found to modulate Integrin Subunit Beta 7 (ITGB7) function, specifically within the t (14, 16) MM subgroup. Choudhury et al. (55) showed that in primary B cells, ITGB7 is down-regulated by H3K27me3 enrichment at the transcription start site and upstream promoter. Of note, MM subgroups involve a complex interaction between DNA methylation and chromatin modification, such as in the t (4, 14) subgroup, where the repressive H3K27me3 mark is substituted by the activating marks H3K4me3 and H3K27ac. This change indicates that MM SET in the t (4, 14) subgroup induces global demethylation of the activating mark H3K36 simultaneously with decreasing trimethylation of the repressive mark H3K27 (56, 57). The t (4, 14) subgroup exhibited *de novo* enrichment of activating histone marks, which was further supported by intermediate DNA methylation levels at the corresponding differentially methylated regions (DMRs) (55). A marked reduction in DNA methylation at enhancer-associated intragenic DMRs, together with increased DHS intensity and enrichment of activating histones and transcription factors, underscores enhanced chromatin accessibility, with a 23 kb CCCTC-binding factor (CTCF)-marked region forming an activation loop that specifically upregulates ITGB7 expression without affecting neighboring genes. Choudhury et al. (55) identified a super-enhancer network on ITGB7 intragenic DMRs in the t (14, 16) subgroup, marked by H3K36me3, H3K4me1, H3K4me3, and H3K27ac. Targeted DNA methylation in MM.1S cells mirrored patient t (14, 16) patterns and increased ITGB7 expression, revealing a nuanced link between methylation and gene expression. sgRNA-2 raised methylation at 5 of 7 CpGs, with CpG-5/6 acting as potential epigenetic switches. Changes in regions 1 and 3 may involve H3K36me3-mediated transcriptional elongation. Choudhury et al. (55) demonstrated that the perturbation of BRD4 in the REN of ITGB7 influences the underlying gene expression, given the functional dependency of epigenetic regulators in these RENs. The results indicated that BRD4 inhibition via JQ1 treatment did not affect endogenous cMAF expression in MM.1S cells (55). Previous studies demonstrating the overexpression or dominant inhibition of cMAF have established the significance of this transcription factor on ITGB7 expression and cell adhesion in MM (58). Furthermore, several genes such as *ITGB7, CCND2, CCR1*, and *Notch* were identified as being regulated by cMAF and MAF candidates (e.g., MAFB) in MM (59). These findings underscore the complexity of super enhancer-driven regulation in cancer, particularly in the context of adhesion molecules such as ITGB7.

### 4.2 Cancer progression and metastasis

The activation of oncogenes is a critical characteristic of cancer, facilitating invasion and metastasis (60). Samples from patients with extensive distant metastases exhibited a reduction in histone H3K9 di- and trimethylation relative to those with regional metastatic cancer (61). A study utilized tumor- and metastasis-derived organoids to investigate the progression of pancreatic ductal adenocarcinoma (PDA), and significant and recurrent alterations in H3K27ac levels were observed predominantly in metastatic organoids, regardless of the metastatic location. Roe et al. (62) also identified the transcription factor FOXA1 as a mediator of enhancer reprogramming that promotes cancer metastasis. These findings indicate a non-genetic mechanism of natural selection in cancer progression, supporting the exploration of epigenetic factors in metastasis. Lysine demethylase 6A (KDM6A) facilitates invasion and liver metastases in Kirsten rat sarcoma virus (KRAS)-mutant PDA mice (54). KDM6A, a member of the COMPASS-like complex, is involved in the demethylation of H3K27me3 and also exhibits functions independent of its enzymatic activity (54). KDM6A induced the mis-localization of the COMPASS-like complex from standard enhancers that regulate cell identity genes to super-enhancers associated with genes governing squamous differentiation and metastasis, such as *ΔNp63, MYC*, and *RUNX3* (54).

Zhang et al. (63) used H3K37ac ChIP-seq data to characterize AJUBA LIM protein (*AJUBA*) as a gene associated with super-enhancers in hepatocellular carcinoma (HCC). The quantity of lung metastasis nodules markedly rose in mice exhibiting elevated AJUBA expression, while it diminished with reduced expression (63). TCF4 interacted with AJBUBA-associated super-enhancers, indicating a potential regulatory role of TCF4 in the oncogenic expression of AJUBA and the metastatic progression in HCC (63). In another study, Kim et al. (64) investigated DNA methylation patterns in gastric cancer to identify novel epigenetic targets. Their study employed RLGS to identify DCBLD2 as a novel epigenetic target in gastric cancer. Overexpression of DCBLD2 results in reduced cell proliferation in 293T and vascular smooth muscle cells (65). Existing evidence indicates that DCBLD2 may significantly influence cancer cell proliferation and metastasis. Kim et al. (64) found that DCBLD2 was often silenced through epigenetic mechanisms in gastric cancer, highlighting its role in suppressing cell proliferation and invasion in this context. Besides, Toyota et al. (66) proposed a novel molecular phenotype characterized by promoter CpG hypermethylation in CRC. This epigenetic silencing correlates with reduced tumor proliferation and invasiveness, underscoring the functional significance of DNA methylation in cancer metastasis. Collectively, these findings highlight the critical role of super-enhancers and DNA methylation in modulating cancer progression and metastasis. Understanding these epigenetic mechanisms offers insights into the metastatic cascade and provides a foundation for developing targeted therapies aimed at disrupting super-enhancer-mediated oncogene activation.

### 4.3 Immune response

**Figure 2:**
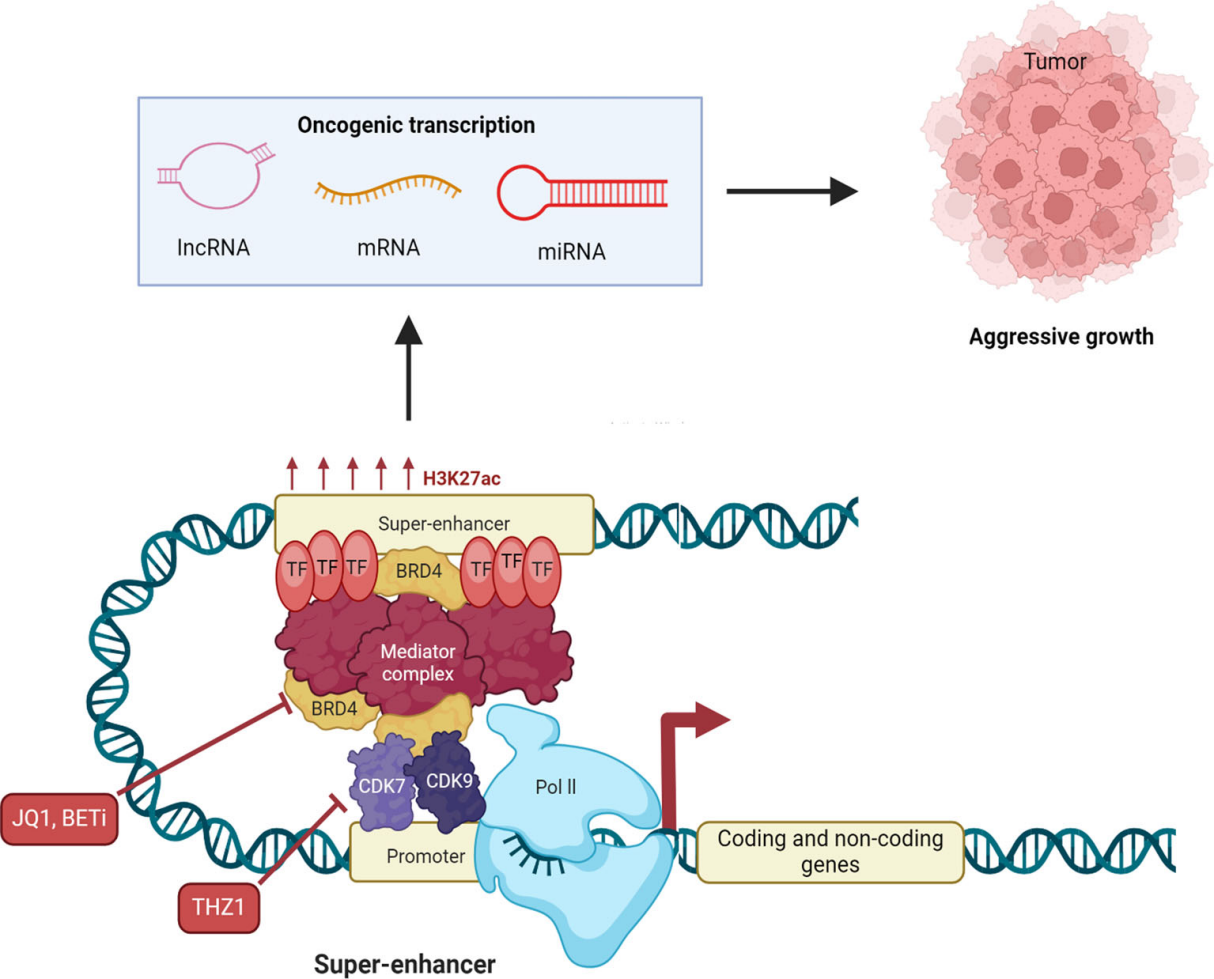
Therapeutic potential of targeting super-enhancers in cancer. Super-enhancers control oncogene expression by high densities of transcription factor binding, histone acetylation, eRNA transcription, and dynamic DNA methylation states. Pathologic DNA methylation at super-enhancers may silence tumor-suppressor programs (by hypermethylation) or hyperactivate oncogenes (by hypomethylation), pointing to their therapeutic potential. supra-enhancers’ eRNAs have enhancer-promoter loop stability and enhance transcriptional output and therefore control by them could be of interest in the clinic. Disruption of super-enhancer function has been the target of pharmacologic intervention. Bromodomain and extraterminal (BET) inhibitors (BETi), such as JQ1, inhibit BET proteins (histone acetyl-reader master transcriptional super-enhancer regulators) and thereby suppress oncogene expression and cancer cell growth. Cyclin-dependent kinase 7 (CDK7) inhibitors disrupt transcription initiation and elongation and block super-enhancer-controlled oncogenic programs. BET and CDK7 inhibitors each exhibit vigorous preclinical and clinical activity in a broad range of cancers. At the same time, epigenetic drugs like DNA methyltransferase inhibitors (DNMTis) are also explored to reverse physiological DNA methylation in super-enhancers and modulate eRNA function, providing additional therapies. All these modalities together are found to have the potential to reverse super-enhancer-driven stacked layers of histone modification, DNA methylation, and eRNA expression in cancer.

Immune modulation by DNA methylation in cancer involves epigenetic changes in super-enhancers and enhancers that regulate genes controlling immune response, immune checkpoint molecules, and inflammation (67). Dysregulated DNA methylation at these enhancer regions can lead to either immune suppression or immune activation, which significantly influences cancer progression and immune escape (68). Chronic lymphocytic leukemia (CLL) is known by the relentless accumulation of CD19+ B cells, leading to its designation as a malignancy that currently lacks a definitive cure (69). The challenges in obtaining curative treatments for CLL are partially influenced by the adaptability of the transcriptional response governed by epigenetic mechanisms. In a study conducted by Shul et al. (69), the researchers aimed to unravel the complexities of the transcriptional landscape in CLL through an integrative approach. Their analysis included B cell enhancer and super-enhancer signatures identified from H3K27Ac ChIP-seq data (CD19+ B cells, GM12878, and MEC1), DNA methylation profiles derived from reduced-representation bisulfite sequencing of samples from CLL patients and healthy donors, and expression patterns obtained via RNA sequencing of CLL and healthy donor samples. Shul et al. (69) identified super-enhancers in each ChIP-seq profile, accounting for approximately 4% of the total enhancers detected. Specifically, they found 741 super-enhancers in GM12878, 374 super-enhancers in MEC1, and 523 super-enhancers in the CD19+ B cells (69). Molecular Signatures Database (MSigDB) gene ontology analysis indicated that numerous genes associated with super-enhancers participate in pathways that regulate immune signaling activation, such as TNF-α via NF-KB and inflammatory response, as well as metabolic homeostasis, including MTORC1 and fatty acid metabolism (69). Additional analysis of the expression levels of super-enhancer-associated genes in CLL patients revealed 190 transcripts that were significantly overexpressed in CLL patient B cells. This overexpressed subset of super-enhancer-associated transcripts was enriched in genes related to immune signaling (such as *FCER2* and *LCK*) and metabolic regulation (such as *ENO2* and *LSR*). Shul et al. (69) analyzed differential DNA methylation from reduced-representation bisulfite sequencing samples, revealing 744 DNA methylation CpG sites that overlapped with identified B cell enhancers. The majority of the DNA methylation CpG sites in CLL exhibited significant hypomethylation (69). Hypomethylated enhancers included super-enhancers associated with overexpressed transcripts *SEPT9*, *ENO2*, *RXRA*, and *CCR7*, along with a typical enhancer linked to the overexpressed transcript Programmed cell death protein 1 (PDCD1). Shul et al. (69) investigated the impact of targeting enhancer-driven gene expression in CLL by comparing the effects of the BET bromodomain inhibitor JQ1 and the cyclin-dependent kinase-7 (CDK7) inhibitor THZ1 (**Figure 2**). Using insights from integrative analyses of B cell enhancers, they performed *in vitro* assays and RNA sequencing on CLL cell lines MEC1 and MEC2 treated with JQ1 or THZ1. The results showed that JQ1 inhibits proliferation in CLL cell lines, suppresses IgM-induced proliferation in primary CLL cells, and modulates the transcription of genes involved in immune signaling pathways. Conversely, THZ1 exhibited distinct effects by reducing cell viability, inducing apoptosis, and selectively downregulating genes linked to metabolic regulation. These findings suggest that DNA hypomethylation in B cell enhancers influences immune signaling and metabolic gene expression in CLL, with JQ1 and THZ1 exerting differential impacts on these pathways through BET bromodomain or CDK7 inhibition.

Me-BAF155 interacts directly with BRD4 to control the expression of oncogenes driven by super-enhancers in Triple-negative breast cancer (TNBC) (70). Furthermore, me-BAF155 inhibits interferon-stimulated gene (ISG) in cancer cells and prevents T-cell infiltration to metastatic locations (71). Kim et al. (70) identified the genomic localization of me-BAF155 at super-enhancers, consistent with the established function of the SWI/SNF complex in regulating super-enhancers across various biological systems. In alignment with the findings of Kim et al. (70), CARM1i significantly suppressed the expression of various super-enhancer-regulated oncogenes, including MYC, similar to the effects observed with JQ1. The clinical application of BET inhibitors has encountered significant challenges, including limited efficacy, pronounced adverse effects, and the frequent emergence of drug resistance in solid tumors (72). Specifically, JQ1 repressed the expression of ISGs, whereas the CARM1 inhibitor activated their expression (73). JQ1 likely promotes metastasis in the 4T1.2 model by suppressing ISG expression in tumor cells. Conversely, EZM2302 enhances ISG activation, increases CD8+ T cell tumor infiltration, and strengthens their cytotoxic function, while leaving CD4+ T cell and macrophage populations unchanged. Kumar et al. (74) discovered CARM1 as a negative regulator of T-cell immunity using a Clustered Regularly Interspaced Short Palindromic Repeat (CRISPR)/CRISPR-associated protein 9 (Cas9) screen. Genetic knockout or pharmacological inhibition of CARM1 in T cells was shown to enhance their anti-tumor activity. These findings suggest that inhibiting CARM1 strengthens the type 1 IFN response within tumors, thereby boosting T cell-mediated immunity and improving anti-metastatic effects. Kim et al. (70) revealed that BAF155 methylation plays a crucial role in the activation of ISGs. In immunocompromised PDX models, CARM1i exhibited pronounced anti-growth and anti-metastatic effects, suggesting that inhibiting CARM1 might also impact the functions of other immune cell types (75).

CXCL13 signaling is recognized for its interference with chemotherapeutic responses, and increased CXCL13 expression by cancer cells enhances autocrine and paracrine effects within the tumor microenvironment (TME), resulting in various outcomes (76). CXCL13 produced by MM cells stimulates the secretion of CXCL13 in adjacent macrophages through Bruton’s tyrosine kinase (BTK) signaling (76). Macrophages subsequently promote CXCL13 expression in MM via transforming growth-factor beta (TGF-β) signaling (77). The CXCR5-CXCL13 axis demonstrates resistance to chemotherapeutic agents, such as bortezomib in MM or 5-fluorouracil in CRC and mantle cell lymphoma (78, 79). The CXCR5-CXCL13 axis facilitates metastasis in breast cancer by regulating the epithelial-to-mesenchymal transition (EMT) (80). CXCR5-CXCL13 signaling facilitates tumorigenesis in phosphatase and tensin homolog (*PTEN*)-deficient cancers through protein kinase C (PKC) signaling (81). The loss of PTEN can lead to the expression of CXCL13 through NF-kB signaling (81). *PTEN* is a gene regulated by p53, indicating that CXCR5-CXCL13 signaling may disrupt p53 regulation. Although CXCL13 is commonly expressed in both hematological and solid tumors, the mechanisms underlying its abnormal expression in cancer cells remain unclear. TGF-β-induced SOX4 enhances CXCL13 expression during Th2 cell differentiation, while retinoic acid and neuronal signaling also stimulate CXCL13 expression in murine embryonic stromal cells (82). Enhancer tethering has been proposed as a critical mechanism for the expression of oncogenes in cancer (83). Gothwal et al. (84) identified a super-enhancer near the CXCL13 locus in cancer cells, linking aberrant CXCL13 expression to impaired GCDBL cell migration and suppression of p53 target genes in B-lymphomas, CRC, and HCC (84). The findings highlight the epigenetic regulation of CXCL13 expression and enhancer-promoter interactions, linking disrupted regulatory mechanisms to the diverse roles of CXCR5-CXCL13 signaling in both hematological and solid tumors.

Cho et al. (85) evaluated the relationship between DMR methylation and immune infiltration by analyzing bulk transcriptome data using CIBERSORT and the LM22 signature matrix. They identified infiltration-associated methylation regions (IMRs), where methylation of specific pDMRs and eDMRs correlated with immune cell infiltration in tumors. Alterations in oncogenic pathways, such as the MAPK and WNT-β-catenin (CTNNB1, WNT3A, WNT7B), were linked to changes in the TME (85). Functional analysis of IMRs overlapping with pDMRs and eDMRs revealed that CpG hypermethylation of immune genes in PMDs contributes to immune evasion and suppression (85). Only 727 of 4,915 IMR-pDMRs and 136 of 6,313 IMR-eDMRs overlapped with PMDs. Reactome pathways, such as ‘GPCR signaling’ and ‘keratinization,’ were highly enriched for genes that are controlled by IMR-pDMRs overlapping PMDs, out of which 12 were immune-related (85). In contrast, cancer hallmarks and cancer genes in CancerSEA and CancerMine were more associated with genes controlled by IMR-eDMRs than by IMR-pDMRs that do not overlap with PMDs. In summary, super-enhancer regulation, particularly through DNA methylation, provides a potential therapeutic avenue, offering insights into immune evasion and immune system activation within the TME. These studies illustrate the multifaceted interplay between enhancer methylation, immune signaling, and immune cell infiltration, highlighting innovative strategies for cancer immunotherapy.

### 4.4 Metabolic reprogramming by super-enhancer methylation in tumors

**Figure 3:**
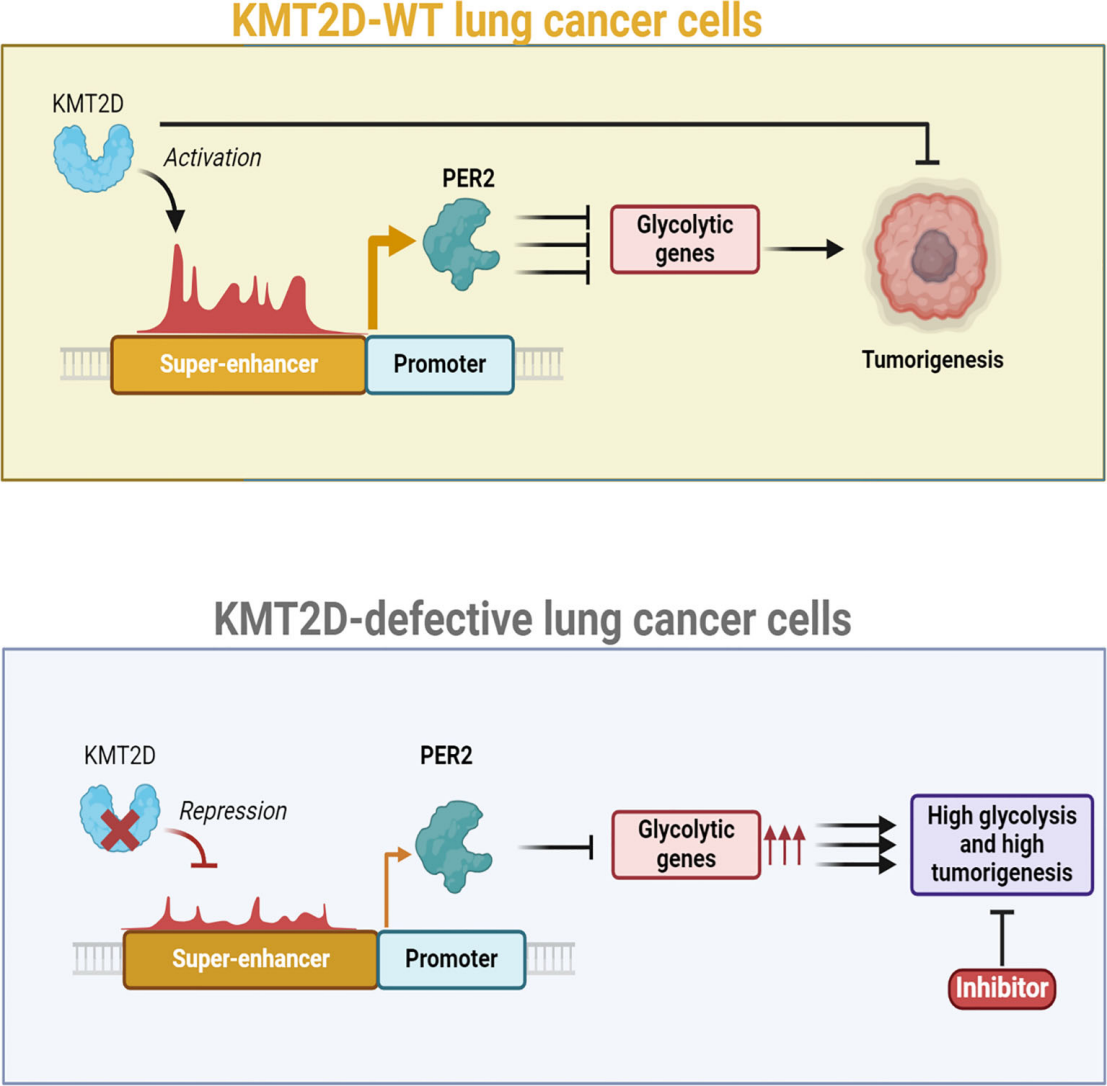
Role of KMT2D as a lung tumor suppressor and its impact on glycolysis in lung cancer, with a focus on super-enhancer DNA methylation. *Kmt2d* loss in lung tissue rapidly promotes tumorigenesis by reprogramming the epigenome and activating pro-tumorigenic signaling, particularly a glycolytic shift. Experimental data show that pharmacologic inhibition of glycolysis selectively inhibits the growth of human lung cancer cells that harbor KMT2D-inactivating mutations, suggesting that such tumors establish a distinct metabolic dependence that may be exploited therapeutically. Mechanistic decomposition suggests that *Kmt2d* deficiency perturbs epigenomic control at critical super-enhancers with a profound impact on the Per2 locus, circadian rhythm repressor. Under normal conditions, *Kmt2d* preserves enhancer activity and histone accessibility through methylation and thus maintains transcriptional activity. Ectopic DNA methylation and super-enhancer structure remodeling ensue on loss of *Kmt2d*, suppressing regulatory signals for effective *PER2* expression. Decrease of *PER2* expression has downstream effects on tumor metabolism. *PER2* is a transcriptional repressor that suppresses expression of several glycolytic genes; loss of *PER2* removes this inhibitory checkpoint and enhances glycolytic flux, thus fueling tumor cell growth and survival. Furthermore, *PER2* deficiency disrupts circadian rhythm–associated control over cell cycle progress, oxidative stress response, and DNA damage repair and further fosters tumor development. Collectively, these studies define KMT2D as a central lung cancer tumor suppressor that functions by maintaining super-enhancer integrity and circadian gene expression. Loss of it, in addition to creating tumorigenesis through metabolic reprogramming, reveals a therapeutic vulnerability: KMT2D-mutant lung cancers are hypersensitive to glycolytic inhibition. On this metabolic vulnerability, or therapies to reconstitute PER2 function, might thus be promising lines of therapy for KMT2D-ablated cancer patients of wider significance than lung cancer.

Metabolic reprogramming is a hallmark of cancer, enabling tumor cells to adapt their energy production and biosynthetic needs to sustain rapid proliferation (86). A pivotal mechanism driving these metabolic changes is the epigenetic regulation of gene expression, particularly through the methylation of super-enhancers. A recent study by Alam et al. (87) revealed that KMT2D acts as an epigenetic regulator in lung adenocarcinoma (LUAD) by promoting the activity of super-enhancers, including the *Per2* super-enhancer, which supports the expression of the tumor suppressor gene *Per2*. Their research showed that KMT2D-driven *Per2* expression suppresses genes involved in tumor-promoting glycolysis (**Figure 3**). Conversely, a deficiency or loss of KMT2D leads to reduced *Per2* levels, resulting in the activation of glycolytic genes. Experiments showed that the heightened glycolysis observed in KMT2D-deficient lung cancer cells could be effectively suppressed through pharmacological intervention (87). The glycolysis pathway is notably enriched in the *Kras; Kmt2d−/−* tumor model compared to the Kras model, as well as in human LUAD tumors exhibiting low or mutant KMT2D relative to those with high wild-type KMT2D (87). Results presented by Alam et al. (87) showed that lung-specific *Kmt2d* deletion accelerates KRAS-driven tumorigenesis in mice, decreases survival, and indicates that *Kmt2d* loss cooperates with oncogenic Kras to promote LUAD progression. The tumor-suppressive role of KMT2D in lung cancer is supported by findings that Kmt2d loss: I) upregulates glycolytic genes (*Eno1, Pgk1, Pgam1, Ldha*, and *Gapdh*); II) downregulates the tumor suppressor gene *Per2*; and III) increases spheroid size in a 3D lung cancer cell culture. Recent studies demonstrate that KMT2D functions as a tumor suppressor in melanoma and pancreatic cancer cells (88, 89). Also, other research has shown that the genetic ablation of *Kmt2d* in B cells increases the development of B cell lymphoma, further supporting the tumor-suppressive role of KMT2D (90, 91). Consequently, the tumorigenic role of KMT2D may vary by cell type, although numerous studies indicate that KMT2D predominantly functions as a tumor suppressor in most tissues.

Recent findings indicate that in medulloblastoma occurring in brain-specific *Kmt2d* knockout mice, the loss of Kmt2d significantly enhances Ras signaling pathways through a marked increase in the expression of various Ras activator genes, including *Rasgrp1, Rasgrf1, Rasgrf2, Rapgef5*, and *Rgl1* (92). The study further demonstrates that KMT2D activates the expression of DNA methyltransferase 3A (*Dnmt3a*), which subsequently represses the expression of Ras activator genes. *KMT2D* knockdown resulted in a reduction of *DNMT3A* expression in human lymphoma cells (90). The *KMT2D* complex has been demonstrated to interact with tissue-specific DNA-binding transcription factors. The *KMT2D* complex co-localizes with MyoD in myocyte differentiation and interacts with Peroxisome proliferator-activated receptor gamma (PPAR-γ) and C/EBP in adipocyte differentiation (93).

Alam et al. (87) identified multiple oncogenic glycolytic genes, including *Eno1, Pgk1, Pgam1, Ldha, Gapdh*, and *Cdk1*, as target genes of PER2. KMT2D-mediated *Per2* activation constitutes a novel tumor-suppressive mechanism that connects an epigenetic tumor suppressor with a regulator of circadian rhythms. PER2 translocates to the nucleus in the evening, where it downregulates gene expression by antagonizing the CLOCK: BMAL1 heterodimeric activator of the circadian rhythm. PER2 undergoes gradual phosphorylation, which facilitates its ubiquitination and subsequent proteasomal degradation. These findings indicate a tumor-suppressive mechanism whereby KMT2D indirectly downregulates glycolytic genes through the enhancement of *Per2* expression via super-enhancer activation, thereby suppressing LUAD. Understanding these mechanisms may pave the way for novel insights into the mechanisms of super-enhancer methylation, which can further define tumor metabolism.

## 5 Clinical and therapeutic significance of super-enhancers in cancer

Super-enhancers are not only mechanistic oncogene expression regulators but also have deep clinical oncology implications (94). Their distinctive epigenetic characteristics and activity-dependent functions make super-enhancers different from typical enhancers, making super-enhancers strong biomarkers and therapeutic targets. Increasing evidence points to the fact that cancer diagnosis, prognosis, and treatment approach can be enhanced by knowledge and targeting of super-enhancers (95). Aberrant super-enhancer activity and DNA methylation profiles represent a molecular signature that marks cancer compared to normal tissue (9). Genome-wide methylation profiling has demonstrated that super-enhancers experience more extreme and cancer-type modifications compared to basic enhancers and thus are extremely predictive of tumor initiation and progression. For example, super-enhancer hypomethylation is frequently correlated with the overexpression of lineage-restricted oncogenes, and this is predictive of tumor aggressiveness and metastatic capability (12, 96, 97). Conversely, super-enhancer hypermethylation linked to tumor suppressor genes has been documented in CRC and breast cancer and holds potential as diagnostic markers for early detection of the disease (98). The methylation alterations relating to super-enhancers can also be used as minimally invasive biomarkers that can be identified using circulating tumor DNA (ctDNA) assays and allow liquid biopsy–based diagnosis (99). The super-enhancer-dependent transcriptional requirement presents a unique vulnerability that is accessible for therapeutic targeting. Super-enhancers are typically bound by coactivators like BRD4, CDK7, and MED1 and are thus ideal targets for pharmacologic disruption (100, 101). BET inhibitors (e.g., JQ1, OTX015) inhibit BRD4 binding at SEs and selectively repress SE-linked oncogenes like MYC and BCL2 (102). Likewise, CDK7 inhibitors (e.g., THZ1) inhibit transcriptional initiation and elongation at SE-mediated loci, with notable efficacy in triple-negative breast cancer (34, 103). Notably, since normal cells depend less on super-enhancer-mediated transcription, therapy with these drugs does have some tumor selectivity that limits systemic toxicity.

Apart from coactivator blockade, epigenetic therapy is also under investigation to reprogram super-enhancer methylation states. DNA methyltransferase inhibitors (DNMTis), such as azacytidine, and histone deacetylase inhibitors (HDACis) can remodel super enhancer function by reactivating access to tumor suppressor–associated super enhancers or silencing oncogenic super enhancers (104). Recent studies suggest that targeted manipulation of super enhancer methylation could resensitize tumors to chemotherapy and immunotherapy, offering a novel strategy for overcoming drug resistance (95, 105). Moreover, CRISPR-based epigenome editing tools are now enabling locus-specific modulation of super enhancer methylation, potentially paving the way for highly precise therapeutic interventions (106). As a result, these findings demonstrate that super enhancers are not merely inactive transcriptional regulators—but rather the hub nodes of oncogenic signaling and immunomodulation. As enhancer mapping at high resolution and single-cell epigenomics continue to improve, super enhancer methylation signatures will inevitably find their place in models of clinical decision-making. Therapeutic approaches in the future could include the treatment of super-enhancer-targeted drugs in conjunction with immunotherapy or metabolic reprogramming therapy, thus leveraging the multifaceted function of super-enhancers in cancer biology. In summary, super enhancers are not only mechanistic drivers but also accessible targets for clinical translation in cancer biology. The prognostic significance, biomarker potential, and treatability of super enhancers make them major players in the new epigenetic oncology paradigm.

## 6 Overview of super-enhancer DNA methylation in the selected cancers

The epigenetic regulation of super-enhancers, particularly DNA methylation, plays a pivotal role in controlling their activity. Aberrant DNA methylation patterns in super-enhancers contribute to oncogenesis by modulating the expression of oncogenes, tumor suppressors, and immune-regulatory genes. In this section, we summarize the findings of super-enhancer DNA methylation in cancer (**Table 2**).

**Table 2:** Overview of super-enhancer DNA methylation on various cancers.

Cancer	Study	Gene	Mechanism	Description	Ref.
Chronic lymphocytic leukemia (CLL)	Clinical	*ENO2, SEPT9, RXRA*, and *CCR7*	FCER2 and PDCD1	Hypomethylation in the super-enhancers of B cells triggered the upregulation of immune and metabolic genes, which are crucial for the progression of CLL.	(69)
Acute lymphoblastic leukemia (ALL), acute megakaryoblastic leukemia	Clinical	*RUNX1*	**-**	In Down syndrome (DS), the *RUNX1* locus was found to be hypermethylated, especially within a super-enhancer specific to hematopoietic stem cells (HSCs). The methylation level of the *RUNX1* super-enhancer was significantly higher in DS compared to control samples.	(107)
B lymphoma	*In vitro*	Chemokine (C-X-C motif) ligand 13 (CXCL13)	CTCFs	MBD1-mediated DNA methylation of super-enhancers played a crucial role in repressing *CXCL13* expression. This regulation was influenced by stress conditions, CTCF, and the DNA methylation status of the promoter, emphasizing the complex interaction of DNA modifications in controlling immune-related genes in cancer.	(108)
CLL	Clinical	*BCL2*	H3K27ac	In CLL, super-enhancers located near critical genes like *BCL2*, *LEF1*, and *CTLA4*, which are involved in lymphocyte proliferation and differentiation, exhibited elevated levels of H3K27ac.	(109)
CLL	Clinical	*CD5, CLLU1*, and *IRF2*	**-**	DNA methylation specific to CLL, especially in class A and C CpG sites, was enriched within super-enhancer regions. These super-enhancers were categorized into “stable” (shared with normal B cells) and “gained” (newly acquired in CLL).	(110)
Multiple Myeloma (MM)	Clinical	*ARID5A*	CTCF	Super-enhancer-CTCF loops at H3K27ac-enriched differentially methylated regions (DMRs) were responsible for regulating the overexpression of specific genes or gene clusters.	(111)
MM	Clinical	*ITGB7*	H3K36me3	Targeted induction of DNA methylation at intragenic enhancers, such as those in *ITGB7*, promoted gene expression, likely through interactions with other epigenetic modifications like H3K36me3. Induced methylation at DMRs overlapping with super-enhancer regions in *ITGB7*, leading to a marked increase in gene expression.	(55)
Colorectal cancer (CRC), Breast cancer, Glioblastoma, Lung cancer	Clinical	*MYC* and *RNF43*	Transcription factor (FOXA2, FOXP1, and FOXQ1) binding	DNA methylation changes in super-enhancers, influenced by histone modifications such as H3K27ac and H3K4me1, were associated with cancer-specific alterations. These methylation changes contributed to the silencing of tumor suppressor genes like *MIRLET7* and *RUNX1*, while promoting the activation of oncogenes such as *MYC* and *RNF43*.	(13)
Gastric adenocarcinoma	Clinical and *in vitro*	*CLDN4* and *ELF3*	ABLIM2, SLC1A2	Super-enhancers with somatic gain exhibited hypomethylation, while those with somatic loss showed hypermethylation. This was confirmed by analyzing loci like *ABLIM2* and *SLC1A2*, highlighting the link between super-enhancer methylation changes and cancer progression.	(50)
Oropharyngeal carcinoma	Clinical	*SMAGP*, and *GPR107*	**-**	Hypermethylation of super-enhancers was linked to the suppression of tumor suppressor genes, such as the reduced expression of SMAGP, a gene important for epithelial cell adhesion.	(112)
Oesophageal squamous cell carcinoma (OSCC)	Clinical	*ZFP36L2*	H3K27ac	The study observed frequent hypermethylation of the *ZFP36L2* super-enhancer region in OSCC. Methylation at specific CpG sites was negatively associated with the expression of *ZFP36L2*, suggesting epigenetic silencing of the gene.	(113)
Nasopharyngeal carcinoma	*In vitro* and *in vitro*	*TRIB1*	R-loop	In cell lines like K562 and GM12878, the super-enhancer regions near *TRIB1* exhibited hypomethylation, while the exons of the TRIB1 gene showed hypermethylation. The hypomethylation in the super-enhancer regions was linked to R-loop formation, which in turn triggered gene activation.	(114)
Lung cancer	*In vitro*	*MYC, E2F6*, and *IRF1*	TF binding and RNA polymerase II recruitment	Hypomethylation of G4 structures in super-enhancers, overlapping with CpG islands, enhances super-enhancer activity and gene expression in cancer. In contrast, methylation of these regions reduces their regulatory function, affecting downstream gene activity.	
Leukemia, CRC, Breast cancer, Chronic myeloid leukemia	*In vitro*	*MYC*	CTCF	Methylation of the enhancer-docking CTCF site disrupts CTCF binding, reducing MYC expression and cellular proliferation, highlighting its key role in cancer development.	(115)
Prostate cancer	Clinical	*KRT5*, and *KRT14*	TRIM29, TP63	Methylation of specific CpG sites within super-enhancers was closely associated with epigenetic changes and transcriptional disruptions specific to PRAD.	(116)
Prostate cancer, Breast cancer	Clinical	**-**	H3K27ac, TCF4, YY-1	Super-enhancers exhibited more significant methylation compared to regular enhancers, with the H3K27ac mark still present.	(12)
Breast Cancer	Clinical	*USF1, SOX4*, and *MYBL2*	Core transcriptional regulatory circuitry	Super-enhancer regions had lower methylation levels than random genomic areas, with cancer samples showing even lower methylation compared to normal tissues. ChIP-seq analysis revealed that super-enhancer regions were enriched with active chromatin marks (H3K4me1, H3K4me2, H3K27ac, EP300), transcriptional marks (H3K4me3), and exhibited greater chromatin accessibility compared to random regions.	(98)
Breast cancer	Clinical and *in vitro*	*ESR1, ERBB2, FBLN2, CEBPA*, and *FAT4*	**-**	Key hypomethylation sites in enhancer regions and hypermethylation sites in CpG islands (CGIs) were identified as regulators of critical genes. These included oncogenes ESR1 and ERBB2, as well as tumor suppressor genes FBLN2, CEBPA, and FAT4.	(117)
Breast cancer	Clinical	*ADM2, TGFBR2, JUN, EGFR*, and *GATA3*	H3K27ac	Several differentially methylated sites (DMS) were found in breast-tissue-specific super-enhancer regions, including the SE-ID-36299 and SE-ID-30649537 regions, which contained 49 and 45 DMS, respectively.	(118)
Breast cancer	Clinical	*HOXB2*	H3K27ac	Hypermethylation at the CpG site cg20401567, situated downstream of the HOXB2 gene, was linked to reduced expression of HOXB2 and other genes in the HOXB family.	(119)
Breast, Myeloid leukemia, MM, Acute promyelocytic leukemia (APL), nasopharyngeal carcinoma (NPC), plasmacytoma tumor, urothelial cell carcinoma (UCC), uveal melanoma	*In silico*	*MIXL1, BLK, PLEKHA2, ACY3, PTPRCAP, TBC1D10C, PLEKHA2, HAND2, HPGD, EHD3, CD163L1, CD27, LPAR5, RBP5, KCNN3, PBXIP1*, *CCDC152, LOC153684, ERMN, C16orf54, CORO1A, ITGAL, LOC606724*, and *MAPK3*	**-**	The study identified 159 differentially methylated super-enhancers, with 87 actively regulating 150 genes. Pathway analysis showed these genes were associated with carcinogenesis in nasopharyngeal, breast, melanoma, and bladder cancers, and were regulated by the epigenetic landscape in these cancers.	(112)
Lung cancer	Clinical and *in vitro*	*SFTPA2, SMDP4, SFTPD*, and *SFTA3*	–	Hypomethylated and hypermethylated F-seDMRs were enriched in the Reactome pathways for “keratinization” and “surfactant metabolism,” respectively. This suggests that enhancer region methylation, rather than promoter methylation, is more influential in regulating tumorigenesis and immune infiltration in lung squamous cell carcinoma (LUSC).	(85)
Lung cancer	Clinical and *in vitro*	*Cytohesin 1 Interacting Protein (CYTIP), TNF superfamily member 8 (TNFSF8, Programmed cell death protein (PD)-1*	–	The hypomethylation of DMRs in *CYTIP* and *TNFSF8* was found to be a stronger predictor of response to anti-PD-1 treatment, as well as progression-free survival (PFS) and overall survival, compared to *PD-L1* expression.	(120)
Lung cancer	*In vitro*	*MYC, E2F6*, and *IRF1*	TF binding and RNA polymerase II recruitment	Hypomethylation of G4 structures in super-enhancer-associated CGIs boosts super-enhancer activity and gene expression in cancer, while methylation reduces their regulatory impact on gene activity.	(121)
Lung cancer	Clinical	*NEUROD1*, and *MYC*	**-**	Genes covered by these super-enhancers, such as *NEUROD1*, *FOXA1/2*, and *NKX2-1*, exhibited a negative correlation between gene body methylation and gene expression.	(122)
Lung cancer	*In vivo*	*KMT2D*	**-**	Loss of *Kmt2d* notably reduced enhancer and super-enhancer activity, as evidenced by a global decrease in H3K4me1 and H3K27ac signals, without affecting H3K4me3 and H3K27me3 levels.	(87)
Lung cancer	Clinical	*SFTPA2, SFTA3*, and *SFTPD*	**-**	The findings highlight the critical role of super-enhancer methylation in tumor progression in LUSC, particularly by downregulating genes related to surfactant metabolism and keratinization, suggesting that super-enhancer methylation could be a key epigenetic mechanism driving tumorigenesis.	(85)
Pancreatic cancer	*In vivo*	*ΔNp63, MYC*, and *RUNX3*	KDM6A	Downregulation of *UTY* is linked to CpG island methylation or Yq11 deletions. Additionally, changes in H3K4me1/H3K27ac and an increase in super-enhancer size, especially in knockout females, suggest that KDM6A regulates super-enhancer delimitation via the COMPASS complex.	(54)
Blader cancer	Clinical	*PI3K-Akt*	lncRNA	The study found a connection between DNA methylation changes and lncRNA expression in bladder cancer, specifically showing reduced 5mC levels in the super-enhancer regions of lncRNA genes in tumor tissues.	(123)
Retinoblastoma	Clinical and *in vivo*	*Ascl1*, and *Crx*	H3K27Ac	During retinal development, super-enhancers associated with genes like *Ascl1* and *Crx* exhibited dynamic changes in activity and DNA methylation. These super-enhancers were identified as crucial regulators of genes that control retinal progenitor cell maintenance and differentiation into photoreceptors.	(124)
Glioma	Clinical	*DICER1*	CTCF	The findings reveal that abnormal methylation of super-enhancers and associated regulatory elements, marked by altered 5hmC and 5mC levels, plays a crucial role in cancer. These epigenetic changes impact key pathways, including WNT signaling and RNA regulation, driving tumor development and progression.	(125)
Neuroblastoma	*In vitro*	*MYCN*	CTCF	Core Regulatory Circuit-driven super-enhancers surrounding *MYCN* demonstrated active regulation through hypomethylation.	(126)
Neuroendocrine carcinoma (NEC)	*In vitro*	*ELF3*	ASCL1	The DNA methylation status of super-enhancer regions was found to regulate *ELF3* overexpression in ASCL1-positive NEC, emphasizing an epigenetic mechanism over transcriptional regulation by ASCL1.	(127)
Hepatocellular carcinoma (HCC)	Clinical	*HGS, CEP131, MAFG, MAFG-DT, FOXK2*, and *SIRT7*	**-**	A total of 2,051 aberrant enhancer-associated DEGs were identified, with upregulated genes linked to cell cycle, DNA repair, and replication, and downregulated genes associated with immune response and metabolism.	(128)

### 6.1 Hematologic malignancies

In hematological malignancies, super-enhancer DNA methylation emerges as a pivotal mechanism in leukemogenesis and disease progression, driving dysregulated transcriptional programs. In this regard, acute myeloid leukemia (AML) clones exhibited aberrant DNA methylation, particularly at CpG sites associated with enhancer regions, characterized by a notable prevalence of hypomethylation (129). AML exhibiting distinct cytogenetic and mutational characteristics demonstrates varying DNA methylation profiles. Notably, mutations in the *DNMT3A* and *IDH* genes display opposing patterns of enhancer DNA methylation, indicating that the epigenetic ramifications of these mutations may significantly influence their malignant phenotype (129). AML with *CEBPA* silencing is an exception, showing promoter hypermethylation with minimal changes at enhancers, reflecting the distinct clinical and biological features of this subtype (129).

Research by Qu et al. (130) indicated that both AML patients with gene mutations and those without have an abnormal DNA methylome when compared to healthy CD34+ cells, with significant alterations observed in enhancer regulatory areas. Genome-wide analysis of these cells has associated alterations in DNA methylation with the deposition of chromatin marks in enhancer regions, demonstrating a substantial association between DNA hypomethylation and active chromatin marks (H3K4me1, H3K4me3, DNase sensitivity, and H3K27ac) (130). Thus, DNA demethylation activates novel and poised enhancers in AML, resulting in a leukemia-associated transcriptome in these cells (130). Significantly, abnormal enhancer DNA methylation in AML has been demonstrated to be independent of the anticipated differentiation-related alterations at these loci, indicating that this atypical DNA methylation profile is distinctive to the pathological condition in AML and may represent a pivotal event in leukemogenesis (131).

A study carried out by Yang et al. (132) uncovered the function of enhancer DNA methylation in AML pathogenesis and its linkage to DNMT3A, a gene that is commonly mutated in myeloid neoplasms. Heterozygous knockout of *DNMT3A* and FLT3-ITD mutation resulted in *de novo* AML through DNA methylation deficiency in DNMT3A knockout mice. Enhancer methylation changes were noted on *DNMT3A* knockdown, with possible sites for transcription factor binding engaging in myeloid differentiation. The same enhancer hypomethylation signatures were found to be present in AML patient samples with the DNMT3A R882 mutation. Genes near these hypomethylated enhancers were enriched for hematopoietic development pathways, enriched strongly for the *HOXB* gene cluster (132). Additional evidence highlighting the significance of enhancer regulatory regions in AML pathogenesis arises from a condition known as enhancer hijacking, characterized by recurrent translocations involving enhancer elements within the myeloid compartment. Patients with AML exhibiting inv (3) or t (3, 3) demonstrate the relocation of the GATA-binding protein 2 (GATA2) enhancer to the EVI1 locus (133, 134). This results in a dual effect of improper EVI1 overexpression alongside GATA2 downregulation, established contributors to AML formation (133, 134).

Of note, a direct correlation between the abnormal suppression of super-enhancers and leukemogenesis has been established in only a limited number of studies (38). Tatsumi et al. (38) revealed that the sustained suppression of GFI1 super-enhancer by LSD1 was crucial for the maintenance of erythroleukemia cells. Moreover, as this repression may be readily reversed by the LSD1 inhibitors NCD38 and NCD25, LSD1-mediated suppression of super-enhancers presents a prospective target for erythroleukemia. While the DNA methylation state in the CA remained mostly unchanged among these cells, it has been shown that the combination of LSD1 inhibitors and the DNA hypomethylating drug, 5-azacytidine, resulted in enhanced efficacy against AML (135). The postponed reduction of CD235a and slight growth inhibition in UT7-EPO may be attributed to the activation of alternative super-enhancers, such as ERG-SE, since Tatsumi et al. (38) previously established that ERG super-enhancer activation by NCD38 facilitated the reduction of CD235a.

Emerging research on cancer has also demonstrated the role of heritable epigenetic modifications in cancer evolution (109). Pastore et al. (109) found a comprehensive examination of the CLL epigenetic landscape with respect to the intraleukemic variety of epigenetic and transcriptional factors. At the H3K27ac regulatory areas, they found substantial chromatin rewiring that was mediated by certain families of transcription factors, namely the NFAT and TCF/LEF families. Pastore et al. (109) demonstrated through the targeted bisulfite sequencing capture test that these regulatory regions exhibited the most significant DNA methylation alterations. In addition, the DNA methylation-RNA mutual information is significantly lower in CLL samples compared to normal B cells, which show coordinated epigenetic transcriptional control leading to higher pairwise mutual information. Pastore et al. (109) analyzed matched DNA methylation and mRNA single-cell data, revealing a greater increase in single-cell mutual information in CLL compared to normal B cells, facilitating direct exploration of this phenomenon. This finding lends credence to the idea that intra-leukemic epigenetic diversity is partially to blame for the modest role of promoter DNA methylation in explaining transcriptional variability in bulk cancer studies (136).

Pastore et al. (109) simulated the combinatorial patterns of histone modifications, DNA methylation, and gene expression to broaden the evaluation of epigenetic coordination beyond epigenetic levels. In particular, genes tagged with H3K27me3^hi^/H3K4me3^low^/H3K27ac^low^ tend to have a consistent transcriptional output in normal B cells, whereas in CLL, the expression levels of these genes are highly unpredictable (137). The therapeutic potential of targeting enhancer methylation is highlighted by emerging strategies combining DNA hypomethylating agents and chromatin modifiers, such as LSD1 inhibitors, to disrupt leukemogenic transcriptional networks. These findings emphasize the critical role of super-enhancer regulation in hematological malignancies and its potential as a therapeutic avenue.

### 6.2 Breast cancer

The intricate patterns of super-enhancer DNA methylation in breast cancer underscore their pivotal role in tumor biology, influencing both oncogene activation and tumor suppressor gene silencing. Aberrant DNA methylation at super-enhancers, characterized by hypomethylation in enhancer regions and hypermethylation in regulatory regions such as CpG islands (CGIs), is a hallmark of breast cancer progression. Jin et al. (117) indicated that most DNA methylation changes in breast cancer are located in the gene body region rather than the promoter region, with the 3’ UTR exhibiting hypomethylation in breast tumor tissue. Hypermethylation in TSS1500, 5’ UTR, and first exon regions diminishes gene expression in breast tumor tissue (117). In breast cancer, upregulated genes are predominantly CGI hypermethylation-independent, while upregulated genes are predominantly associated with enhancer hypomethylation, implicating divergent gene silencing and activation mechanisms (117). Indeed, CGI hypermethylation represses *FBLN2*, *CEBPA*, and *FAT4* expression, while enhancer hypomethylation enhances *ERBB2* and *ESR1* expression (117). Besides, Heyn et al. (13) found that 14% of the super-enhancers examined experienced alterations in CpG methylation in their corresponding human tumors, such as normal breast tissue compared to breast cancer. The predominant DNA methylation alteration, a reduction in CpG methylation, was identified in 75% of cases, whereas 25% of super-enhancers exhibited an increase in DNA methylation in cancer samples (13). The hypomethylation events were notably unspecific, correlating with the general loss of DNA methylation typically seen in cancer samples, with the exception of CRC, where they were highly specific to super-enhancer loci (13). Consequently, to identify functional epigenetic modifications, Heyn et al. (13) opted to concentrate on the hypermethylated events, which were prevalent in genes linked to transcriptional and metabolic processes as well as angiogenesis. Significantly, hypermethylation was corroborated using DNA methylation microarray analysis in a distinct cohort of 714 primary cancer cases, wherein 58.1% of the examined DNA hypermethylation at super-enhancers was validated. The findings indicate that the hypermethylation identified in the cancer cells reflects modified DNA methylation patterns at super-enhancer regions in primary tumors (13). Heyn et al. (13) determined that copy number variations (CNVs) did not bias the primary cancer samples, as they identified substantial differences in DNA methylation levels between normal and CNV samples in only a negligible proportion of the super-enhancers. Notably, through oxidative bisulfite treatment combined with DNA methylation analyses, they eliminated the possibility that the increase in DNA methylation found in cancer was attributable to an elevation of 5-hydroxymethylation, a particular cytosine modification that interferes with 5-methylation in bisulfite-based analyses and was found to be enriched in conventional enhancer regions (13). To clarify the functional implications of the observed cancer-specific super-enhancer DNA methylation alterations, they examined the effect of tumor-associated increases in super-enhancer DNA methylation on gene expression. Similar to the proximal regulatory gene regions, which are known to exhibit a general repressive effect due to DNA methylation, Heyn et al. (13) identified a correlation between increased DNA methylation in breast super-enhancer regions and the repression of associated genes in MDA-MB-468PT cell lines. They confirmed the association between super-enhancer hypermethylation and the transcriptional silencing of the related genes across several breast tumor types. Heyn et al. (13) demonstrated that super-enhancers were influenced by their CpG methylation in normal cells and specific aberrant DNA methylation events in cancer target them, potentially affecting the expression of downstream genes. They proposed that localized alterations in transcription factor binding influence super-enhancer DNA methylation patterns, therefore affecting target gene expression. Consequently, the levels of DNA methylation at super-enhancers signify regulatory activity and additionally identify associated transcription factors. In cancer, the altered expression of critical transcription factors creates new super-enhancers that promote oncogene expression. This phenomenon was partially elucidated by identifying FOXQ1 as a potential factor influencing the differential DNA methylation at CRC super-enhancers and the overexpression of significant oncogenes, including RNF43 and *MYC*. Heyn et al. (13) underscore that the creation of comprehensive databases of DNA methylomes at base resolution might enhance the comprehension of the regulatory roles of DNA methylation beyond the extensively examined proximal promoter gene areas.

Yang et al. (118) comprehensively examined the methylation landscape of super-enhancers in The Cancer Genome Atlas (TCGA) BRCA cohort and identified differential methylation sites (DMS) associated with super-enhancers, demonstrating significant efficacy for risk classification of BRCA patients. Alterations in the distribution of super-enhancer DNA methylation in cancer cells correlate with the aberrant activation or repression of transcription in the respective target genes (20, 96). The acquired DNA methylation indicates that transcription factors influence the localized activity of super-enhancers, and the modulation of the DNA methylation profile by trans-acting factors affects the transformation process in carcinogenesis (96). Consequently, Yang et al. (118) found that the DMS in the super-enhancer regions could serve as an indicator for patient prognosis. The CPM derived from various methylation sites in the super-enhancer regions can assess the prognosis of breast cancer. Abnormal DNA methylation in the promoter region of an oncogene or tumor suppressor gene is characteristic of tumors; these aberrant methylations contribute to the tumorigenesis and spread of breast cancer (138, 139). This suggests that alterations in methylation status within enhancer regions of BRCA facilitate tumorigenesis and progression, underscoring the significance of DNA methylation localization in the development of cancer biomarkers.

### 6.3 Lung cancer

Super-enhancer DNA methylation represents a pivotal mechanism in the epigenetic regulation of lung cancer, influencing the expression of oncogenes and tumor suppressor genes through alterations in enhancer activity. Researchers investigated the epigenetics of SCLC by tracing the locations of lactotransferrin (LTF) binding sites, enhancers dependent on H3K27ac histone modification, and promoter DNA methylation (122). Using the SCLC cell line databases (https://discover.nci.nih.gov/SclcCellMinerCDB/), Pongor et al. (122) integrated whole-genome DNA methylation, enhancer H3K27ac ChIP-seq, and ChIP-seq for main LTFs driving SCLC pathogenesis (NEUROD1, ASCL1, and POU2F3) to expand the understanding of gene regulation in SCLC and discover the potential value of the MethylationEPIC 850k array. Algorithms were programmed to automatically measure the amounts of methylation in the promoter and gene bodies, copy number, and promoter acetylation for every gene (122). They also showed how each epigenetic marker predicts gene expression by analyzing their chromosomal distribution (122). Moreover, Pongor et al. (122) found that enhancer regions have lower DNA methylation levels and that genic regions encapsulated by super-enhancers are often absolutely hypomethylated. The increased promoter mark H3K4me3, which prevents the deposition of DNA methylation to broader genomic areas in super-enhancers, is likely associated with this (140, 141). The profile of genes covered by super-enhancers, like *FOXA1/2*, *NEUROD1*, and *NKX2*-1, was shown in this unexpectedly negative connection between expression and gene methylation (122). In line with the super-enhancer analysis, phylogenetic analysis of the H3K27ac findings for the enhancers with the highest signal intensity range across cell lines revealed that the cell lines clustered into three main groups, distinguishing the NEUROD1, ASCL1, and POU2F3 subtypes and validating the unique enhancer signatures of the SCLC subtypes (142). Based on the subtypes, four primary clusters were identified in the cluster heatmap of the H3K27ac signals (122). Clusters 1, 2, and 3 were shown to have an abundance of DNA sequence motifs from ASCL1, POU2F3, and NEUROD1, according to the HOMER sequence motif analysis (122). Recent efforts have also been made to apply this categorization to clinical samples (143, 144). These subtypes can be grouped according to their super-enhancer activity, according to recent basic investigations (122). Using variable enhancers, rather than only super-enhancers, also revealed the clumping. It was expected that there would be minimal overlap between the ASCL1, NEUROD1, and POU2F3 binding sites, given that each subtype has its own increased enhancer signal. Furthermore, ASCL1, NEUROD1, and POU2F3 binding sites had reduced DNA methylation levels (145). Lastly, Pongor et al. (122) showed that levels of promoter enhancer signals are powerful gene expression predictors, even more so than DNA methylation and copy number obtained from DNA methylation.

Given the significant impact of enhancer methylation on carcinogenesis in LUSC, Cho et al. (85) postulated that super-enhancers play crucial roles in the epigenetic regulation of cancer progression. Super-enhancer DMRs (seDMRs) were established based on the overlap between annotated super-enhancers and DMRs. Cho et al. (85) identified around 1000 seDMRs with an equal number of hypomethylated and hypermethylated areas and approximately 1500 target genes. They found <200 functional seDMRs (F-seDMRs) and Reactome pathways for “keratinization” and “surfactant metabolism” showed substantial enrichment for target genes of hypomethylated and hypermethylated F-seDMRs, respectively. Interestingly, all five enhanced Reactome pathways among hypermethylated F-seDMR targets were linked to surfactant metabolism. Cho et al. (85) discovered that two hypermethylated F-seDMRs target genes encoding surfactant protein A2, surfactant-associated protein A3, and surfactant protein D. These findings indicate that hypermethylation of super-enhancers may be a significant epigenetic regulatory mechanism for driving tumor growth in lung tissue. Overall, Cho et al. (85) conducted a functional enrichment analysis and found that enhancer methylation, rather than promoter methylation, plays a significant role in carcinogenesis and immune infiltration. This finding implies that enhancer methylation plays a significant role in canonical cancer pathways and neoplastic features unique to LUSC as compared to other forms of cancer. For example, disturbance of the keratinization and pulmonary surfactant pathways has been shown to alter clinical outcomes, mostly for LUSC31,35-37, and these parameters were shown to be related to eDMR targets in the current investigation. These results suggest that disruption of these pathways may be mediated by abnormal methylation of enhancers in LUSC. Taken together, studies on SCLC and LUSC have revealed that super-enhancers are often hypomethylated in genic regions, allowing for subtype-specific enhancer activity that drives oncogenic pathways. Conversely, hypermethylation of super-enhancers, such as those regulating surfactant protein genes, underscores their role in tumor progression and immune evasion.

### 6.4 Gastrointestinal cancer

The methylation state of super-enhancers is a key regulator of gene expression and cell fate, particularly in gastrointestinal (GI) cancer contexts such as CRC (146, 147). Super-enhancers are critical in driving the expression of oncogenes such as *HSF1* (148). Ren et al. (148) found that *HSF1* mutations may be partially responsible for *HSF1* overexpression in CRC patients. Overexpression of *HSF1* was associated with several driver genes in CRC, notably TP53, which showed the strongest correlation (148). This aligns with the findings of Isermann et al. (149), who reported that mutp53 can enhance HSF1 activity by disrupting the inhibitory WTp53-HSF1 interaction. Ren et al. (148) then investigated the influence of epigenetic changes (including DNA methylation) on HSF1 expression. Unexpectedly, they discovered a super-enhancer in the TSS of HSF1 mRNA. To further research the reasons for HSF1’s elevated expression in CRC, Ren et al. (148) examined the driver genes, which are important nodes of regulatory networks and signaling pathways. Using the TCGA portal database, they discovered that *HSF1* expression was associated with several driver genes, such as *TP53, APC, KRAS*, and *PIK3CA* (148). In line with this finding, HSF1 expression was substantially related to mutant TP53 in the UALCAN database (148). Aside from genetic changes, tumors are also associated with epigenetic alterations, including histone modifications, non-coding RNAs, and DNA methylation. Using the UCSC database, Ren et al. (148) identified a strong H3K27ac signal at the transcription start site of HSF1. Furthermore, the expression of *HSF1* correlates favorably with that of BRD4, the master reader that binds to acetylated histones and regulates gene transcription. These findings collectively suggested a possible role for super-enhancers in regulating HSF1 expression. Ren et al. (148) also investigated the link between HSF1 and DNA methylation in CRC, and they discovered that HSF1 had decreased levels of DNA methylation in CRC compared to normal tissues. In addition, the cBioPortal database demonstrated a clear negative connection between *HSF1* expression and DNA methylation levels in CRC (148).

Charlet et al. (12) examined the role of DNA methylation in super-enhancers through a comparative analysis with gene promoters in colon cancer by the highly methylated colon cancer cell line HCT116. The recently released 850K MethylationEPIC BeadChip Infinium array incorporates CpG sites in enhancer regions from the Fantom5 and ENCODE projects (150). However, Charlet et al. (12) utilized whole-genome bisulfite sequencing data from their NOMe-seq samples, which demonstrated that the active H3K27ac mark coexists with the typically repressive DNA methylation mark in standard enhancers and across extensive chromatin regions in super-enhancers. These sites likely represent functional elements that regulate gene expression; they serve as binding sites for transcription factors and exhibit the anticipated inverse relationships among DNA methylation, H3K27ac, and accessibility. The findings demonstrated that bivalent loci, characterized by a positive correlation between DNA methylation and H3K27ac, were predominantly located outside transcription factor-binding sites, where chromatin accessibility is reduced (12). The removal of cytosine methylation in DKO1 cells significantly impacts bivalent enhancer structures and results in a marked reduction of the H3K27ac mark. This effect was further validated through DNA demethylation induced by transient 5-Aza-CdR treatment. Charlet et al. (12) indicated that DNA methylation adversely affects H3K27ac levels at enhancers. Currently, it has been indicated that super-enhancers may completely collapse upon the removal of a single H3K27ac-enriched constituent or the absence of a transcription factor (33, 41). Charlet et al. (12) demonstrated that DNA methylation significantly influences both regular and super-enhancer regions upon genetic or transient removal.

Additionally, to clarify the roles of m3Es in CRC, Lin et al. (151) analyzed their distribution by integrating H3K4me3 and H3K27ac ChIP-Seq data. Their findings indicated that m3Es were extensively distributed throughout the human genome and played a crucial role in regulating inflammatory gene expression in CRC. Lin et al. (151) employed a native ChIP approach to eliminate interference from promoter H3K4me3 signals. These findings indicate that m3Es account for approximately 10% of total enhancers, suggesting their widespread distribution in the genome and significant roles in cellular functions. The AP-1/JUN transcription factor is implicated in the regulation of m3E activity and is closely linked to immune pathways and functions as an oncogene in various cancers. Recent epigenomic investigations and motif analyses focused on enhancer profiling have identified and validated the AP-1 family as a crucial group of oncogenic transcription factors (152, 153). Lin et al. (151) have established potential links among AP-1 members, tumor-specific Vm3Es, inflammation, and cancer, highlighting their significant roles in tumorigenesis and metastasis, particularly in the contexts of cancer, immunity, and stress response. In summary, in GI cancers, such as CRC, the interplay between super-enhancers and DNA methylation can emerge as a pivotal area of research, shedding light on tumorigenic mechanisms and potential therapeutic interventions.

### 6.5 Hepatocellular carcinoma

DNA methylation, an epigenetic mechanism widely implicated in gene regulation, has emerged as a critical factor in modulating super-enhancer activity in various cancers, including HCC (22, 154). In HCC, aberrant DNA methylation patterns, particularly hypomethylation, have been identified at enhancer regions, leading to oncogene activation and global transcriptional reprogramming (96, 155). Xiong et al. (9) developed a method combining epigenomic and transcriptomic data to infer enhancer-target interactions, enabling the identification of genes regulated by differentially methylated enhancers in HCC. Their epigenomic study identified a hypomethylated CCAAT/enhancer-binding protein-beta (C/EBPβ) enhancer that promotes HCC tumorigenicity via global transcriptional reprogramming. Xiong et al. (9) discovered widespread hypomethylation of transcriptional enhancers in HCCs by analyzing DNA methylation in primary tumors, nontumor, and normal liver tissues. Their research identified an aberrantly methylated enhancer of prognostic importance, which forms a positive circuitry with its target gene to confer HCC characteristics such as angiogenesis, proliferation, and invasion (9). Xiong et al. (9) advanced the understanding of the HCC methylome. Although promoter hypermethylation and hypomethylation have been documented in HCC development, their whole-genome bisulfite sequencing (WGBS) analysis revealed a notable decrease in methylation across most differentially methylated elements (DMEs). Through nanoscale chromatin profiling of HCC tissues, they precisely mapped enhancers and confirmed enhancer hypomethylation-associated C/EBPβ overexpression (9). This was validated using bisulfite pyrosequencing and quantitative reverse transcription polymerase chain reaction (qRT-PCR) on paired tumor and non-tumor HCC samples. Other HCC-related genes have been found, including SRC tyrosine kinase and ATG7, an autophagy-related pro-survival gene. This investigation discovered new enhancer-hypomethylated and over-expressed targets, including IFNGR2 and SLC45A4, which can play essential roles in HCC due to their involvement in controlling hepatitis B virus (HBV) viraemia and redox homeostasis (156). The HBx oncoprotein, which has been demonstrated to cause demethylation of distal regulatory areas, could be a contributing component to HCC tumorigenesis (9). The HBx TG HCC model recapitulates the C/ebpβ enhancer regulatory network, showing the role of HCC risk factors in enhancer dysregulation during carcinogenesis. Xiong et al. (9) indicated that C/EBPβ enhancer hypomethylation reactivates eRNA, resulting in C/EBPβ transcription. This transcription then binds to and activates its enhancer, creating a self-reinforcing cycle. They found a 3-kb eRNA that was unidirectionally transcribed for *C/EBPβ* gene regulation. Knocking down this eRNA reduces *C/EBPβ* gene reactivation by DNA demethylation, as well as inhibiting HCC cell proliferation and invasion. The findings of Xiong et al. (9) support the functional significance of other tumor-specific eRNAs, including androgen- and estrogen-dependent eRNAs in prostate and breast cancers, which play crucial roles in gene transcription. This epigenomic analysis highlights the tumorigenic role of the C/EBPβ enhancer in HCC. Deleting this enhancer significantly reduced HCC tumorigenicity, leading to genome-wide co-depletion of *C/EBPβ* and *BRD4* occupancy, as well as severe dysregulation of gene expression. C/EBPβ functions at both promoters and enhancers, but the findings suggest that C/EBPβ/BRD4 complexes primarily target enhancers to regulate gene activation in HCC cells. Absence of the C/EBPβ enhancer leads to the loss of C/EBPβ/BRD4 from thousands of enhancers, resulting in decreased H3K27ac levels and gene expression. In summary, the function of super-enhancer DNA methylation in HCC highlights the intricate interplay between epigenetic modifications and oncogene activation. The hypomethylation of super-enhancers, as evidenced by the C/EBPβ enhancer, serves as a pivotal driver of HCC tumorigenicity through the reactivation of oncogenic transcriptional programs and enhancer remodeling.

### 6.6 Nervous system neoplasms

Super-enhancers in nervous system cancers are often modified through mechanisms, including DNA methylation, histone modifications, or interactions with specific TFs (95). Dhar et al. (92) found that some super-enhancers repressed medulloblastoma and described a novel tumor-suppressive mechanism in which MLL4, an H3K4 methyltransferase, was required to maintain wide H3K4me3 and super-enhancers at tumor suppressor genes. Specifically, Xu et al. (157) employed ChIP-seq to analyze H3K27ac, a marker of active chromatin, in glioblastoma (GBM) tissues, oligoastrocytoma, normal brain samples, and cell lines. This comprehensive mapping of active regulatory regions in GBM uncovered tumor- and subtype-specific enhancer-gene interactions, transcription factor networks, and oncogenic dependencies (157). Investigating differentially regulated active regulatory elements (AREs), particularly super-enhancers, provided insights into oncogenic pathways, molecular classification, and epigenetic mechanisms underlying GBM subtypes. The findings of Xu et al. (157) can help researchers better understand the epigenetic pathways that underlie both normal brain functions and GBM. According to their objective study of AREs and gene expression in main tissues, there is a favorable association between total enhancer signals and target gene expression. Current molecular classification relies heavily on gene expression markers; however, these data show that there is extra heterogeneity in GBM at the ARE level (157). Research on super-enhancers that regulate gene expression in a context-dependent manner has revealed tumor- and subtype-specific regulation of target genes, such as *RFX2* and *TGIF1*, long non-coding RNAs (lncRNAs) such as MIR99AHG and LINC01094, and druggable targets, such as BRD4, MKNK2, and WEE1 (157). Despite the fact that the exact cell type that gives rise to each GBM tumor is still a mystery, animal genetic studies have shown that neural stem cells, early progenitors, astrocytes, and neurons can undergo oncogenic transformation into malignant gliomas (158). GBM-specific super-enhancer targets have an overrepresentation of TFs, which indicates a thorough reorganization of the transcriptional network. Consistent with this, TF-centric super-enhancer signature genes isolated from GBM may reliably categorize glioma patients according to tumor grade, survival rate, and other molecular characteristics in patient cohorts. A study by Xu et al. (157) suggests that therapeutic drugs targeting BET bromodomain proteins could disrupt fundamental regulatory circuits, offering a potential therapeutic strategy. Additionally, they have documented that the BET protein degrader ZBC260 exhibited potent anti-GBM action. Furthermore, Xu et al. (157) identified a wide range of functional targets and pathways driven by super-enhancers that are associated with subtype specificity, highlighting promising avenues for future research on the dependencies of actionable tumors. The ARE dataset serves as an optimal resource for conducting in-depth biological investigations, particularly concerning lncRNAs, and for testing related hypotheses. The findings of Xu et al. (157) lay the groundwork for developing more precise scoring systems that can effectively map the involvement of super-enhancer domains and their targets to specific subtypes.

Furthermore, to examine the relationship between super-enhancers and high 5hmC loci, Azizgolshani et al. (125) utilized super-enhancer coordinates identified in various brain-derived cell lines. While 5-hydroxymethylation in pediatric brain tumors has been explored, existing studies have not achieved genome-scale analysis at locus resolution (125). An association between 5hmC and anaplasia was identified through immunohistochemistry analysis of 5hmC across all classifications of brain tumors (159). Wu et al. (160) recently demonstrated that elevated levels of 5hmC correlate with poorer survival outcomes in pediatric posterior fossa ependymomas. An analysis at the nucleotide level revealed enrichment of high 5hmC loci in genes essential for normal craniofacial and neurodevelopment, thereby reinforcing the association between these tumors and developmental neurobiology (161). These findings indicate that differentially hypohydroxymethylated CpGs are enriched in molecular pathways commonly associated with childhood brain tumors, especially those involving WNT signaling and β-catenin binding. Accumulation of 5hmC at 5′ splicing sites in the exon–intron boundary has been proposed as a connection between this epigenetic marker and alternative splicing (162). Azizgolshani et al. (125) indicated that the high 5hmC sites are localized to 5′ untranslated regions, consistent with prior research. Furthermore, these loci are enriched in genes associated with the posttranslational regulation of gene expression, including *DICER1*, *AGO2*, and *EIF2C2*. The relationship between 5hmC and CTCF, a methylation-sensitive transcription factor associated with alternative splicing and RNA polymerase II regulation, has been documented in embryonal cells. Elevated levels of 5hmC are associated with diminished nucleosome binding to DNA and decreased CTCF attachment (163). 5hmC oscillates at 150 nucleotides, corresponding to the length of nucleosome-wound DNA, and is proposed to function as a linker that binds CTCF to DNA (164). In summary, the current study presented the first genome-wide cytosine-specific analysis of 5hmC in three categories of nervous system cancers: embryonal, glioma, and ependymoma. Taken together, the findings show that super-enhancers are pivotal in shaping the transcriptional and epigenetic landscapes of nervous system neoplasms.

## 7 Mechanistic diversity of super-enhancer DNA methylation in a number of cancer type

Although super-enhancer methylation is a common feature in many malignancies, its mechanistic roles differ considerably across cancer types due to variations in transcriptional networks, epigenetic landscapes, and oncogenic signaling dependencies (165). For example, in squamous cell carcinomas (SCCs), super-enhancer dynamics are tightly linked to lineage-defining transcription factors such as *TP63* and *SOX2*, which frequently reside near super-enhancer regions (166). Aberrant methylation in these loci disrupts enhancer-promoter interactions, reprograms keratinocyte identity, and drives EMT, thereby contributing to aggressive tumor phenotypes. Furthermore, enhancer hypomethylation in SCCs promotes the activation of oncogenes involved in cell proliferation and invasion, reflecting a unique vulnerability of this tumor type to enhancer reprogramming. In contrast, breast cancer demonstrates a distinct set of mechanistic alterations, where enhancer hypomethylation often amplifies estrogen receptor (ER)-dependent signaling pathways. Super-enhancer methylation changes at loci such as ESR1 and ERBB2 can upregulate hormone-driven oncogenic transcriptional programs (167, 168). Conversely, hypermethylation of super-enhancers linked to tumor suppressor genes (e.g., *FBLN2*, *CEBPA*) contributes to silencing critical homeostatic pathways. These opposing patterns highlight the dual role of enhancer methylation in breast cancer, with hypomethylation driving oncogene activation and hypermethylation reinforcing tumor suppressor gene silencing.

The mechanistic diversity across cancers may therefore be explained by three interrelated factors. First, tissue-specific transcription factor occupancy dictates the dependency of certain enhancers on DNA methylation status, as different transcription factors establish unique regulatory programs in distinct tumor types (169). Second, chromatin accessibility landscapes vary across cell types, influencing the susceptibility of super-enhancers to methylation changes and thereby shaping their regulatory potential (170). Third, oncogenic signaling pathways—such as Wnt/β-catenin in CRC, estrogen receptor–driven signaling in breast cancer, and squamous lineage programs in squamous cell carcinomas—selectively exploit enhancer methylation to sustain malignant transcription (171). All of these examples illustrate that, although super-enhancer methylation is a unifying characteristic among heterogeneous cancers, its functional significance is extremely context-specific and mirrors the interaction between cellular identity, chromatin structure, and oncogenic signaling. Taken in totality, these findings suggest that although aberrant DNA methylation at super-enhancers is a unifying cancer hallmark, its impact is extremely context-specific and mirrors cancer-type–specific vulnerabilities that can inform targeted therapy.

## 8 Super enhancer DNA methylation of cancer to diagnostic and therapeutic implications

Dysregulated DNA methylation was the first epigenetic alteration found in tumors, and it typically disrupts signal pathways and plays a role in the onset of several diseases (172). A number of tumor cells, including ovarian, breast, colon, and cervical cancer cells, have been studied for abnormal DNA methylation, which has been linked to the expression of specific oncogenes or tumor suppressor genes (173, 174). Consequently, DNA methylation was employed as a biomarker for early cancer detection. Differentially methylated enhancers may potentially reveal information about the differentiation status of malignancies because enhancer methylation levels are closely related to cell differentiation (175). The prognosis of patients may be influenced by differential enhancer methylation since less differentiated cancers are generally more aggressive and metastatic than more differentiated neoplasms (176). Recent research by Hu et al. (177) examined methylation alterations throughout the genome in renal cell carcinoma (RCC). According to their findings, the downregulation of target genes was associated with altered methylation, which was especially enriched in kidney-specific enhancers (177).

The tumor methylome status was expected to impact the therapeutic response to cancer immunotherapy, particularly Programmed cell death protein 1 (PD-1) inhibitor-based therapy, since DNA methylation alters disease states through epigenetic regulation of gene expression (120). Cho et al. (120) found more than 1,400 differential methylated chromosomal regions between responders and non-responders to anti-PD-1 treatment and suggested a set of pDMRs and eDMRs that are associated with the response to treatment based on annotated cis-regulatory elements. However, the Infinium 450K methylation array, which is the basis for most published tumor methylomes available in the public domain, examines only a small subset of the CpG sites in distal regulatory elements (120). To compare, the Methylation EPIC Array (EPIC chip) measures ~850,000 CpG locations, including more than 90% of the 450K coverage and a further 350K sites in enhancers (120). Enhancers are enriched for disease-associated variants and are essential for the spatiotemporal regulation of gene expression. Therefore, determining enhancer areas for methylation-based epigenetic control is crucial to comprehending the course of the disease and the effectiveness of treatment. Cho et al. (120) used the regulatory target genes of DMRs, whose roles are better understood, to ascertain their functional impact. They discovered by pathway enrichment analysis of these target genes that the epigenetic regulation of the anti-PD-1 response through DNA methylation was linked to immune-related, carcinogenic, and metabolic-regulatory pathways. These findings confirm that the identified DMRs and their target genes are reliable, as these pathways have a role in tumor immunomodulation. Surprisingly, Cho et al. (120) found that eDMRs, not pDMRs, primarily controlled the immunomodulatory pathways in the anti-PD-1 response. More thoroughly, they showed that DMRs in the anti-PD-1 response contain enhancers for human leukocyte antigen (HLA) genes, which encode important antigen presentation components, and discovered that these HLA eDMRs overlap with super-enhancers, which are extremely active enhancers that play important roles in defining cellular properties (120). These eDMRs might be viable therapeutic targets for enhancing the anti-PD-1 response since a number of therapies that target disorders involving super-enhancers are undergoing clinical trials (178).

Of note, the Jumonji C (JmjC) domain histone H3K27me3 demethylase KDM6A is a member of the KDM6 family, which also includes KDM6B, which is encoded by an autosomal gene, and UTY, which is encoded by the Y chromosome (54). In order to control developmental pathways, KDM6A inhibits the Polycomb Repressive Complex 2 (PRC2)-mediated H3K27 tri-methylation, which is catalyzed by the methyltransferase EZH2 (54). Nevertheless, *Kdm6a* deletion in mice showed that a number of developmental processes were mainly unaffected by the demethylase activity. Additionally, KDM6A is a crucial part of the COMPASS (COMplex of Proteins Associated with Set1)-like complex, which includes KMT2C or KMT2D methyltransferases in addition to the core proteins WDR5, RBBP5, DPY30, and ASH2L. These methyltransferases mono-methylate H3K4 to delimit enhancer chromatin, which is why the complex is called COMPASS (54). H3K27ac-marked enhancer clusters generate super-enhancers, which often become hijacked during neoplastic transformation and tend to activate genes that coordinate cell destiny and lineage commitment (54). The most often mutated epigenetic regulators in cancer, including pancreatic cancer, are KDM6A, KMT2C, and KMT2D. Andricovich et al. (54) discovered that *KDM6A* loss activated a super-enhancer that regulates ΔNp63, MYC, and RUNX3 and rewired enhancer chromatin. While RUNX3 is a newly discovered driver of metastasis, ΔNp63 and MYC drive squamous differentiation and are molecular indicators of poor prognosis (54). The observations demonstrated that JQ1 alone was sufficient to produce a therapeutic effect in *KrasG12D; Kdm6a* null mice and COMPASS-defective PDA cell lines, in contrast to a prior publication that demonstrated synergy of JQ1 with either HDAC inhibitors or gemcitabine in *KrasG12D*; *Trp53* null mice (54). In addition to altering the neoplastic cells, JQ1 decreased the number of fibroblasts linked to cancer that expressed αSMA and inhibited the desmoplastic response. Andricovich et al. (54) reported that gene signature loss caused by Kdm6a is linked to IL-6 signaling, an essential pro-inflammatory pathway involved in triggering desmoplasia, while studies increasingly shift toward identifying the growth factors and cytokines that orchestrate the desmoplastic microenvironment. Super-enhancer reprogramming is progressively becoming a required process in metastatic pancreatic cancer, such as the pancreatic progenitor and squamous-like subtypes. Although these subtypes employ distinct super-enhancer reprogramming processes that activate separate pathways, they exhibit a molecular susceptibility to BET inhibitors. Because BET inhibitors restore cell identity and make tumors more sensitive to existing treatments, they may be a promising adjuvant therapy for metastatic pancreatic cancer.

## 9 Conclusion

Super-enhancers in cancer biology drive the expression of oncogenes and other cancer-specific genes, creating canonical gene expression signatures in cancer cells. Our current understanding of the genetic basis of malignancy, therefore, informs our perspective on current notions about super-enhancer methylation. The integration of epigenetic and genetic data specific to particular cancers in individual patients will identify the precise nature of the malignancy more accurately. Tumor-type super-enhancer landscapes can be interrogated to identify novel oncogenes and the regulators of their expression. Super-enhancers play a role in squamous cell carcinomas and breast cancer, but it is a mechanism for other cancers. There are suggestions in GI cancer, brain neoplasm, breast cancer, esophageal squamous cell carcinoma, and pancreatic cancer where super enhancer-associated genes are enriched in EMT and metastasis. The findings indicate that the level of CpG methylation in normal cells identifies locally active regulatory domains at super-enhancers, which are targeted by cancer-specific aberrant DNA methylation events with the potential to affect the expression of downstream target genes. The findings also highlight the value of creating more extensive catalogs of human DNA methylomes at base resolution to gain a deeper understanding of the regulatory roles of DNA methylation beyond the best-characterized proximal promoter gene regions. Due to their involvement in cancer, therapeutically targeting super-enhancers is of significant interest. There are several BET inhibitors of hematologic malignancies and solid tumors that are in different stages of clinical trials. Inhibition of CDK7 represses transcription of super-enhancer-activated oncogenes in osteosarcoma and esophageal squamous cell carcinoma. Treatment with a CARM1 inhibitor reduces me-BAF155 and represses genes regulated by an oncogene super-enhancer, triggering the interferon response. Targeting CARM1 methylation of BAF155 with a CARM1 inhibitor could be a promising treatment, either alone or in combination with BET inhibitors, or in cases of BET-resistant disease. Our understanding of super-enhancer landscapes in cancer has grown significantly in the past several years.

Surprisingly, the interplay between super-enhancers and DNA methylation in cancer is a relatively new field of research, but it is still with some core knowledge gaps. Although DNA methylation is known to influence super-enhancer function, the actual functional consequence is yet to be clarified. Super-enhancer locus methylation can lead to transcriptional suppression or dysregulation of gene expression; however, the causal mechanisms and consequences for downstream gene expression remain unresolved. Moreover, only a limited number of cancer-type-specific super-enhancers have been described to date because comprehensive chromatin profiles have not been achieved in most tumor types. The existing research has a tendency to extrapolate evidence from homologous cancers and thus may not adequately consider the variation in the super-enhancer landscape of particular tumor types. The other challenge is understanding the hijacking process of super-enhancers, where chromosomal translocations relocate SEs to oncogenes and thus drive their overexpression. Despite this being described, the exact processes and their tumorigenic role have to be better understood. Furthermore, the heterogeneity of activity of super-enhancers, both within and between tumors, makes it challenging to interpret their functional roles in regulating processes such as metastasis and treatment response. The integration of multi-omics data, including transcriptomics, epigenomics, and proteomics, is critical to fully comprehend the characterization of super-enhancer function; however, computational tools are currently rarely adequate to fully harmonize such data sets. Lastly, as super-enhancers are new targetable therapeutic targets, the issue of developing selective agents that modify super-enhancer function without affecting normal gene expression is a huge challenge. This limitation must be overcome to reveal new biomarkers, identify oncogenic mechanisms, and drive super-enhancer-targeted cancer therapy.
